# Triphenylphosphine‐Based Covalent Organic Frameworks and Heterogeneous Rh‐P‐COFs Catalysts

**DOI:** 10.1002/chem.202002150

**Published:** 2020-08-28

**Authors:** Yubing Liu, Alla Dikhtiarenko, Naizhang Xu, Jiawei Sun, Jie Tang, Kaiqiang Wang, Bolian Xu, Qing Tong, Hero Jan Heeres, Songbo He, Jorge Gascon, Yining Fan

**Affiliations:** ^1^ Key Laboratory of Mesoscopic Chemistry of MOE School of Chemistry and Chemical Engineering Jiangsu Key Laboratory of Vehicle Emissions Control Nanjing University Nanjing 2100093 P. R. China; ^2^ KAUST Catalysis Center, Advanced Catalytic Materials King Abdullah University of Science and Technology Thuwal 23955 Saudi Arabia; ^3^ Green Chemical Reaction Engineering University of Groningen 9747 AG Groningen The Netherlands

**Keywords:** covalent organic frameworks, heterogeneous catalysts, olefin hydroformylation, triphenylphosphine

## Abstract

The synthesis of phosphine‐based functional covalent organic frameworks (COFs) has attracted great attention recently. Herein, we present two examples of triphenylphosphine‐based COFs (termed P‐COFs) with well‐defined crystalline structures, high specific surface areas, and good thermal stability. Furthermore, rhodium catalysts with these P‐COFs as support material show high turnover frequency for the hydroformylation of olefins, as well as excellent recycling performance. This work not only extends the phosphine‐based COF family, but also demonstrates their application in immobilizing homogeneous metal‐based (e.g., Rh‐phosphine) catalysts for application in heterogeneous catalysis.

## Graphical abstract

**A turn up for turnover**: Two triphenylphosphine‐based COFs (P‐COFs) that stack in an eclipsed AA manner and have high crystallinity, high specific surface areas and good thermal stability have been synthesized. The application of these P‐COFs in phosphorus coordination catalysis was demonstrated by the hydroformylation of olefins over supported Rh/P‐COFs catalysts, which showed high turnover frequencies and excellent recycling.

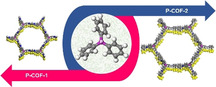

## Introduction

Covalent organic frameworks (COFs), materials with porous crystalline structure assembled by covalent bonds,^1^ have attracted much attention in recent years because of their ordered pore structure, easy functionalization, high stability, and low density. These materials have great potential applications in many fields such as gas storage, separation,^2^ sensing,^3^ energy conversion,^4^ solid‐state ion conducting,^5^ and catalysis.^6^ Since the first report on COFs in 2005,^7^ a large number of structures have been designed and synthesized successfully. Yang et al. proposed a useful genomics method for the high‐throughput construction of COFs and established a library of 130 genetic structural units (GSUs) with a database of about 470 000 materials.^8^ COFs are mainly functionalized by nitrogen‐containing functional groups, such as triazine,^9^ porphyrin,^10^ and Salen.^11^ Very recently, great efforts have been devoted to synthesizing phosphine‐based functional COFs due to the unique features of phosphine, for example, in phosphine organocatalysis.^12^ These attempts used phosphine‐containing building blocks, such as hexachlorocyclotriphosphazene,^13^ triphenylphosphine (PPh_3_),^14^ and dibenzyl phosphite;^15^ however, the obtained phosphine‐functionalized COFs show poor crystallinity^13^, ^14^ and are more likely to be amorphous porous organic polymers (POPs).^15^, ^16^ Conventionally, the planar tripodal sp^2^‐hybridized structural units tend to form a well‐defined layered structure stabilized by π–π stacking interactions.1a, 2a, 17 In contrast, a flexible sp^3^‐hybridized unit, such as triphenylphosphine, tends to undergo a tetrahedral distortion due to repulsion from the lone pair of electrons. This interaction forces the unit to deviate from planarity and twist to a smaller angle (<180°) between phenyl propellers when forming a two‐dimensional structure, resulting in POP materials.^18^ In a paper recently published on‐line, Tao et al.^19^ successfully synthesized a first example of triphenylphosphine‐based COFs, which presents both eclipsed AA stacking and staggered ABC stacking crystals in one sample, owing to the trigonal pyramidal geometry of triphenylphosphine.

**Scheme 1:**
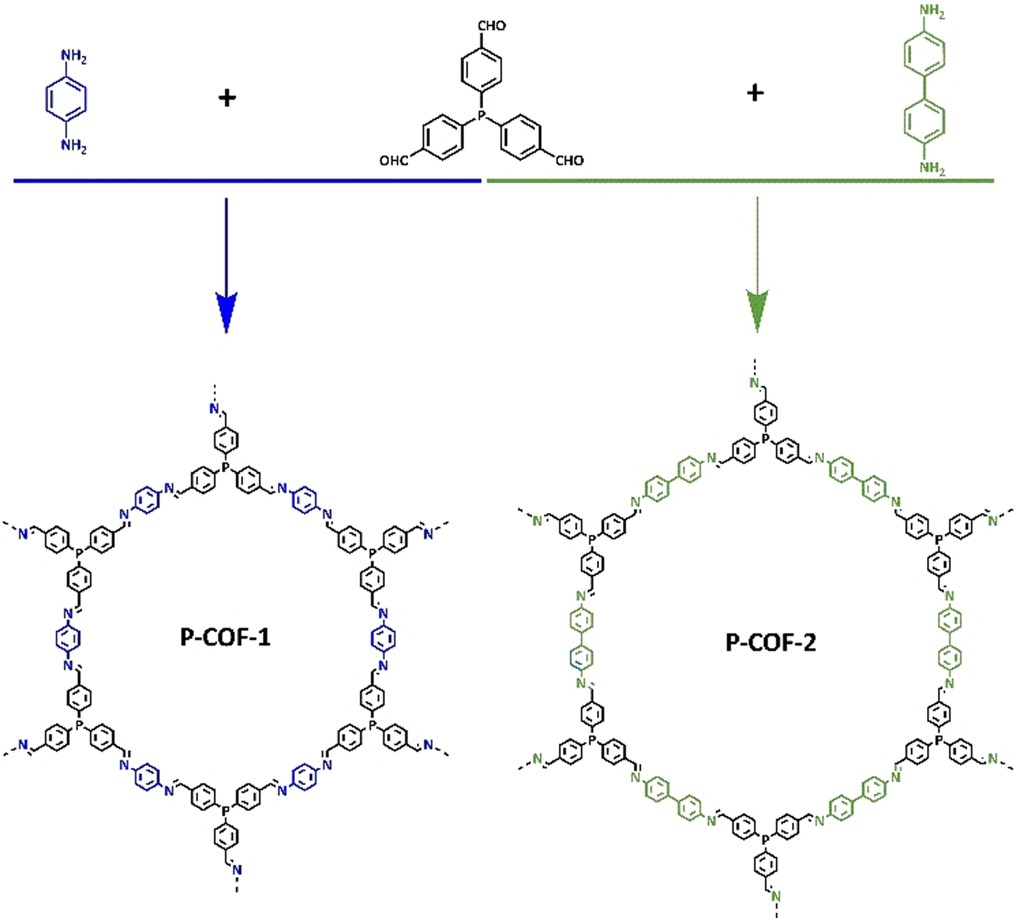
Schematic representation of the synthesis of P‐COFs.

Herein, we report an improved synthesis of two examples of triphenylphosphine‐based COFs (P‐COFs, Scheme 1). These two P‐COFs stack only in an AA eclipsed manner (vide infra) and have high crystallinity and specific surface areas, and good thermal stability. Considering that triphenylphosphine is a very important ligand for organometallic complexes (e.g., homogeneous Rh‐phosphine based catalysts),^12^, ^20^ we further demonstrate here that P‐COFs are superb support materials, acting as promoters (ligands) as well for the immobilization of homogeneous catalysts. Such heterogeneous P‐COF‐supported catalysts (e.g., Rh‐P‐COFs) have shown excellent catalytic activity, product selectivity, and catalyst recyclability for hydroformylation of olefins (vide infra).

## Results and Discussion

**Figure 1:**
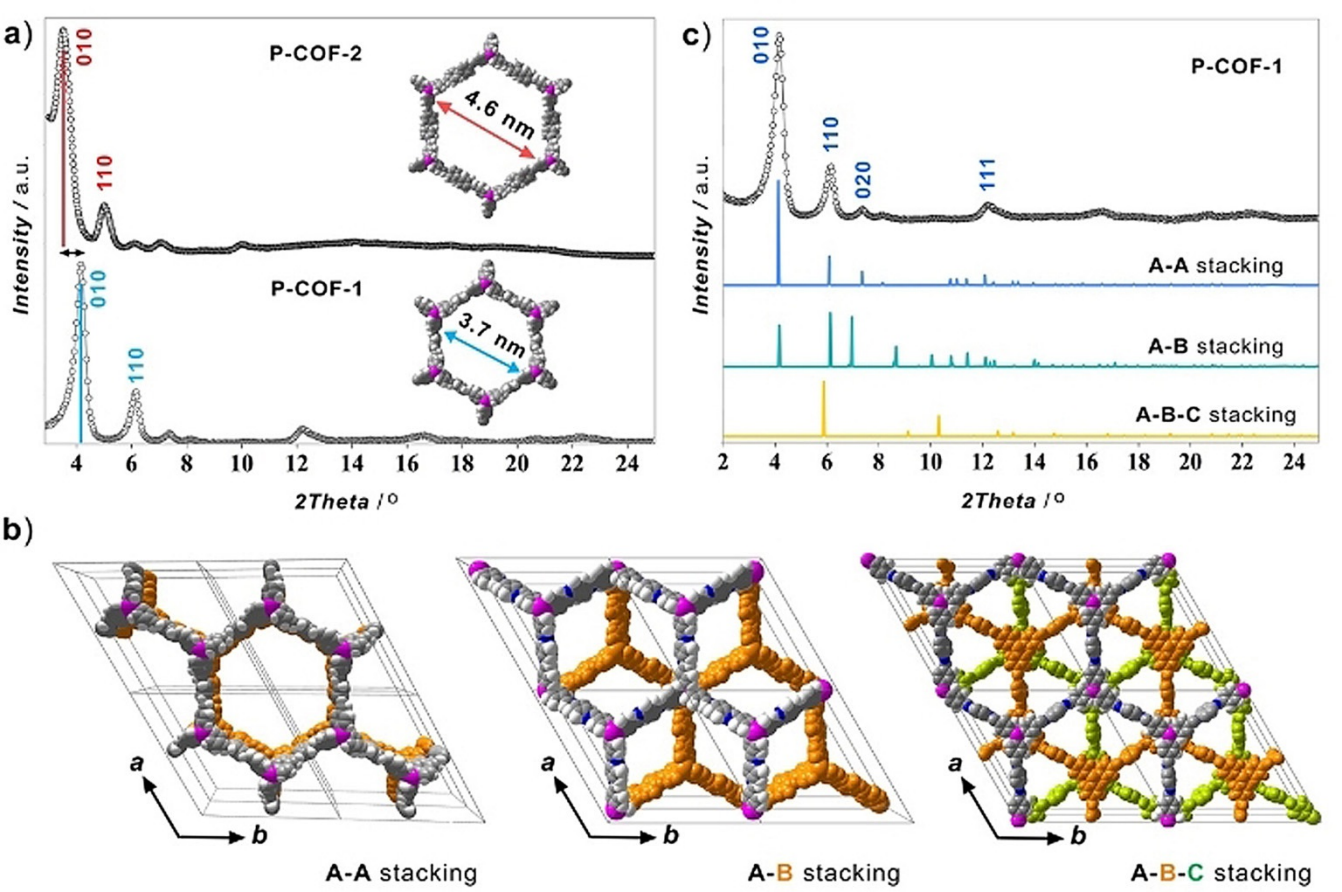
a) Powder X‐ray diffraction patterns of P‐COF‐1 and P‐COF‐2 revealed isostructural AA stacking with different pore sizes. b) The structural arrangements of AA, AB, and ABC stacking in P‐COF‐1 viewed along the *c*‐axes. c) PXRD pattern of P‐COF‐1 (black dots) compared to the simulated for AA eclipsed (blue curve, *P*6 space group), AB staggered (turquoise, *P*6_3_ space group), and ABC staggered‐interpenetrated (orange, *R*‐3 space group) models.

Two imine‐linked P‐COFs were synthesized (Section 2 in the Supporting Information) through a Schiff base reaction of tris‐(4‐formylphenyl)phosphane (TFP, CAS No. 67753‐41‐7, synthesized according to references)^21^ with *p*‐phenylenediamine (PPD, CAS no. 106‐50‐3) or benzidine (CAS no. 92‐87‐5), denoted P‐COF‐1 and P‐COF‐2, respectively (Scheme 1). To gain insight into the crystalline structure of the P‐COFs, powder X‐ray diffraction (PXRD) was performed in combination with simulations. The PXRD patterns of P‐COF‐1 and P‐COF‐2 (Figure 1 a) exhibited an intense peak at 2*θ*=4.2 and 3.4, individually, along with minor peaks at 2*θ*=6.1, 7.3, 8.1, 12.1, 16.1 for P‐COF‐1 and 2*θ*=5.0, 6.1, 7.1, 10.0 for P‐COF‐2. To elucidate the structure of P‐COF‐1 and calculate its unit cell parameters, several possible 2D models with eclipsed and staggered stackings (Figure 1 b and Figure S1 in the Supporting Information) were constructed and optimized applying geometrical energy minimization using a universal force‐field method. Exploration of several possible models (Figures 1 c and S1, Tables S1–S3) confirms that the best fit for the observed peaks, in both position and relative intensities, corresponding to 100, 110, 020 and 111 reflections of the eclipsed structure with an AA sequence of 2D layers. Comparison of experimental PXRD for P‐COF‐1 and P‐COF‐2 (Figure 1 a) clearly evidences their isostructural characteristics. In addition, shifts in peak positions for P‐COF‐2 with respect to P‐COF‐1 are observed, indicating an enlargement of unit cell parameters for P‐COF‐2, as a result of extension of the ring units. Hence, we propose that both P‐COF‐1 and P‐COF‐2 structures stack in an AA eclipsed manner adopting hexagonal P6 settings and forming a system of open 1D pore channels extended along the *c*‐axis. This indicates that the developed syntheses in this submission produce P‐COFs with higher purity, compared with the reported P‐COF‐1^19^ crystals stacked in both eclipsed AA and staggered ABC manners. *Le Bail* refinements confirmed plausible assignment of the space group, as evidenced by the negligible difference between the simulated and experimental diffractograms for P‐COF‐1 (Figure S2) and P‐COF‐2 (Figures S3 and S4) yielding unit cell parameters of a=*b*=32.55 Å, *c*=7.9 Å for P‐COF‐1 (Tables S1) and a=*b*=40.35 Å, *c*=7.9 Å for P‐COF‐2 (Tables S4). The corresponding pore diameters for P‐COF‐1 and P‐COF‐2 were calculated to be 37 and 46 Å, respectively, with an interlayer separation of 7.9 Å.

**Figure 2:**
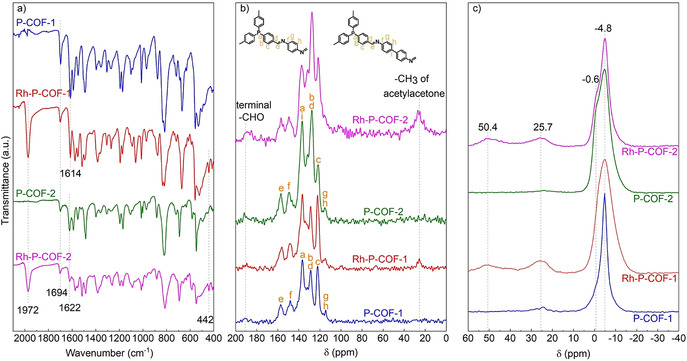
a) FTIR spectra, b) ^13^C CP‐MAS ssNMR, and c) ^31^P static ssNMR of P‐COFs and Rh‐P‐COFs.

The C=N bonds in the P‐COFs are formed by the condensation of aldehyde groups of TFP with the amino groups of *p*‐phenylenediamine or benzidine,^22^ resulting in the disappearance of the N−H bonds, as evidenced by the diminished N−H stretching bands at 3100–3400 cm^−1^ for *p*‐phenylenediamine (Figure S5) and benzidine (Figure S6). A relatively weak C=O stretching band at 1694 cm^−1^ was still present in the FTIR spectra of P‐COFs (Figure 2 a), probably because of the presence of residual terminal aldehyde groups of TFP at the terminal edges of the P‐COFs (Figures S5 and S6).4b, 23 ^13^C CP‐MAS solid‐state NMR spectra of the P‐COFs (Figure 2 b) show characteristic resonances for the C atom of a C=N moiety located at *δ*=156 ppm,^24^ consistent with Fourier transform infrared (FTIR) spectra of the P‐COFs (Figure 2 a, with C=N stretching bands at 1614 and 1622 cm^−1^ for P‐COF‐1 and P‐COF‐2, respectively). ^31^P static solid‐state NMR spectra of the P‐COFs (Figure 2 c) show a single sharp peak at *δ*=−4.8 ppm in line with that for TFP (Figure S8). There is a presence of a little collar around *δ*=−0.6 ppm in P‐COF‐2, which may represent P‐COF‐2 with different polymerization degree from that of *δ*=−4.8 ppm.

**Figure 3:**
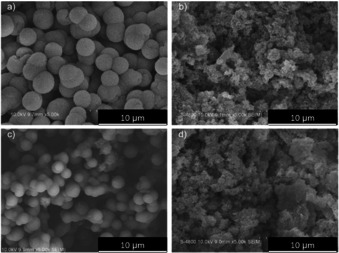
SEM images of a) P‐COF‐1, b) P‐COF‐2, c) Rh‐P‐COF‐1, and d) Rh‐P‐COF‐2.

**Figure 4:**
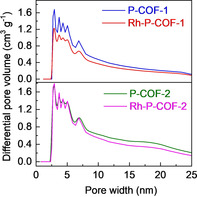
NLDFT pore size distribution from Ar adsorption of P‐COFs and Rh‐P‐COFs.

**Figure 5:**
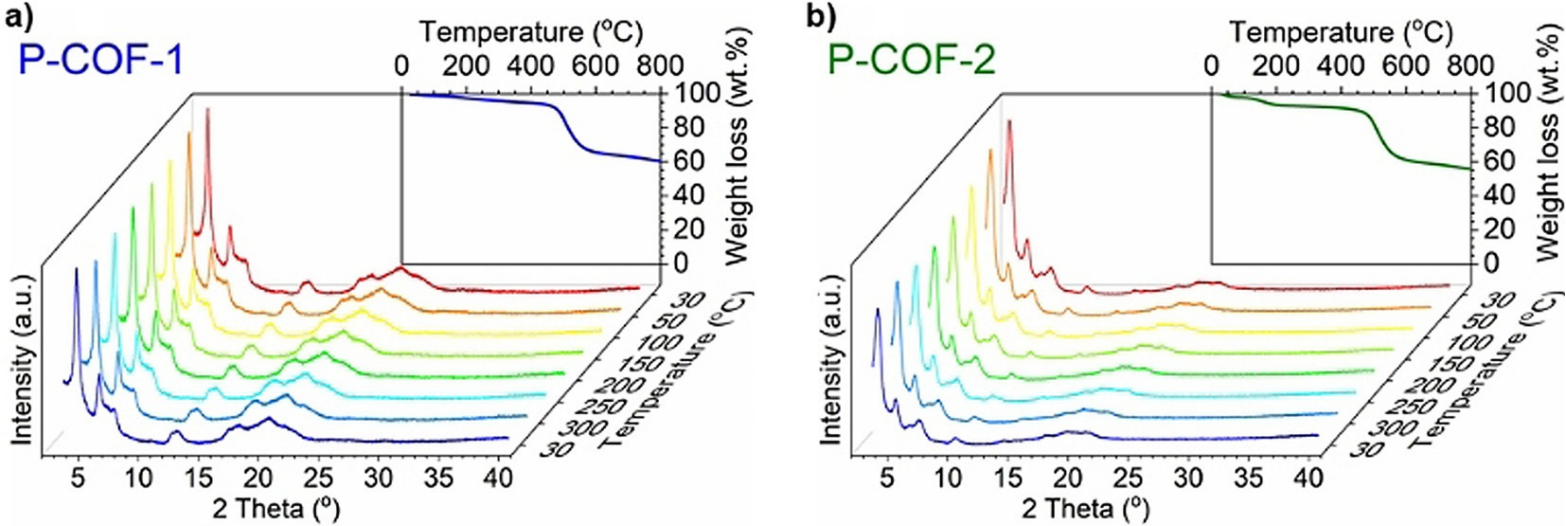
In‐situ VT‐PXRD patterns (under vacuum) and TGA curves (under N_2_) of P‐COFs.

Scanning electron microscopy (SEM, Figure 3) images of P‐COFs show the microspheric crystals with urchin‐like surface morphology, which might be considered as the result of aggregation of a large number of nanosheets (Figure S9) formed by π–π stacking of P‐COF layers.^25^ Adsorption/desorption of Ar and N_2_ (Figures 4 and S11) were performed to characterize the pore structure of P‐COFs. N_2_ adsorption/desorption isotherms of P‐COFs (Figure S10 a) show the combined characteristics of type I and type IV,^26^ indicating the microporous (*P*/*P*_0_<0.1) and mesoporous (0.4<*P*/*P*_0_<1) structure. The pore size distributions (PSDs) of the P‐COFs (Figure 4), calculated on the basis of the nonlocal density functional theory (NLDFT),^27^ show a wide pore distribution over the range of 2–25 nm for P‐COFs. The Brunauer–Emmett–Teller (BET) surface areas based on nitrogen adsorption/desorption isotherms of the P‐COFs are 903 m^2^ g^−1^ (P‐COF‐1, close to the *S*_BET_ for the reported P‐COF‐1)^19^ and 2387 m^2^ g^−1^ (P‐COF‐2); as well as the corresponding total pore volumes are 0.69 cm^3^ g^−1^ (P‐COF‐1) and 4.22 cm^3^ g^−1^ (P‐COF‐2). Notably, these surface areas of triphenylphosphine‐based COFs are higher than those for amorphous triphenylphosphine‐based POPs.^18^ Both P‐COFs show good thermal stability under inert atmosphere, which is evidenced by the negligible weight loss up to 450 °C under N_2_ atmosphere during thermogravimetric analysis (TGA, Figure 5) and the well‐retained crystallinities up to 300 °C under vacuum during the in situ variable temperature PXRD (in situ VT‐PXRD, Figure 5) analysis.^28^ In addition, the P‐COFs are insoluble in water and common organic solvents such as acetone, ethanol, hexane, *N*,*N*‐dimethylformamide (DMF) and tetrahydrofuran (THF). To examine the chemical stability of P‐COFs,^29^ the samples were exposed to different chemical environments for 24 h, including THF, DMF, ethanol, boiling water, HCl (aq) (pH 1), concentrated HCl (4 m), NaOH (aq) (pH 14), and concentrated NaOH (4 m). P‐COF‐1 and P‐COF‐2 retain their original skeleton and crystalline structure after treatment in THF, DMF, and ethanol, as indicated by well‐kept PXRD patterns (Figure S11). Comparatively, P‐COF‐1 has better stability in boiling water and NaOH (aq) than P‐COF‐2. Both P‐COF‐1 and P‐COF‐2 lost their crystallinity after acidic medium treatment, likely related to the hydrolytic nature of the imine bonds, which was also observed for other imine‐linked COFs.6b, 11

**Figure 6:**
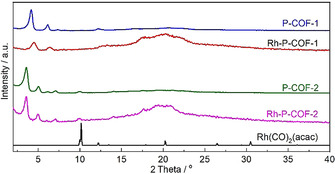
PXRD patterns of P‐COFs, Rh‐P‐COFs and Rh(CO)_2_(acac).

**Figure 7:**
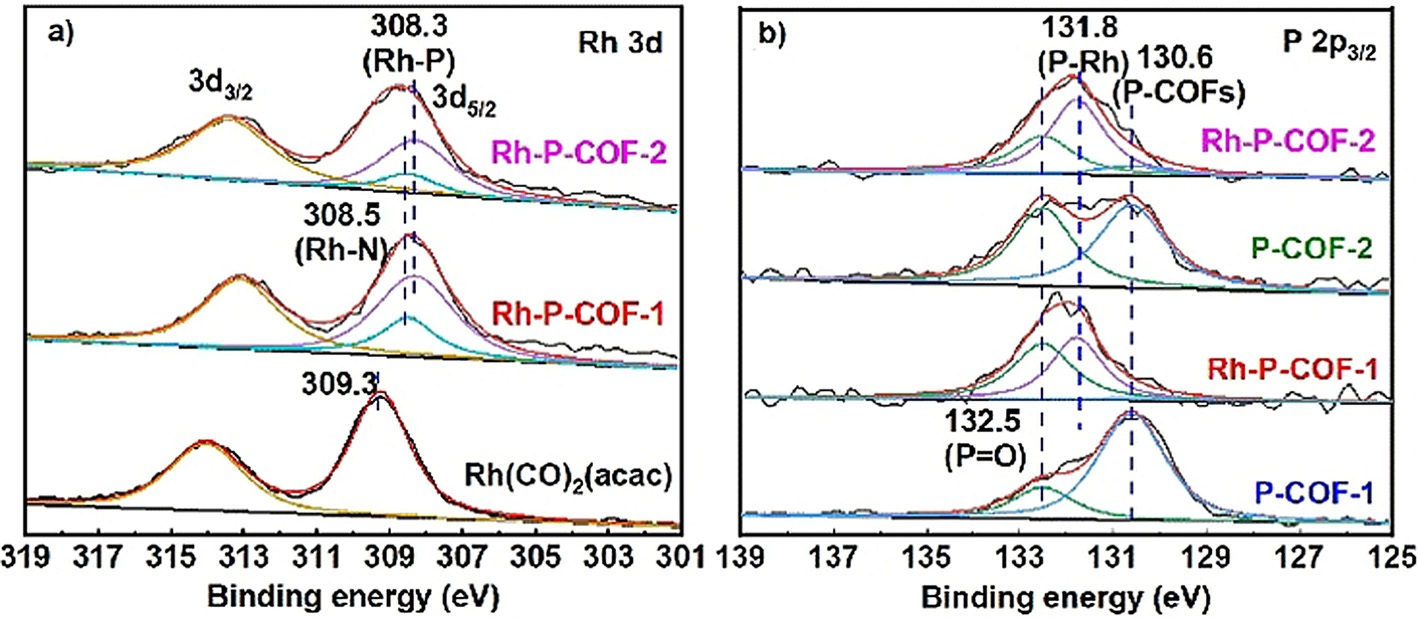
XPS of a) Rh 3d and b) P 2p_3/2_ for Rh(CO)_2_(acac), P‐COFs and Rh‐P‐COFs.

Extending the novelty and promising structures of P‐COFs, both P‐COFs have been used as support materials containing triphenylphosphine ligands for the immobilization of metal complex catalysts. To demonstrate this, an easy post‐treatment (Section S2.3 in the Supporting Information) of two P‐COFs with (acetylacetonato)dicarbonylrhodium (I) (Rh(CO)_2_(acac)) was performed. After Rh loading, the morphology of P‐COFs was maintained, as shown by SEM (Figure 3) and TEM (Figure S9). Those typical diffraction peaks for P‐COFs were preserved, indicated by PXRD (Figure 6). In addition, diffraction peaks for Rh(CO)_2_(acac) were not observed on both Rh‐P‐COFs. However, the lattice fringes of Rh (111) plane with approximately 0.26 nm spacing can be observed on Rh‐P‐COF‐1 treated by NaBH_4_ (Figure S10). These results indicated that the Rh(CO)_2_(acac) is uniformly distributed on Rh‐P‐COFs, in accordance with the SEM‐EDS mapping (Figure S13) of the Rh‐P‐COFs. FTIR spectra of Rh‐P‐COFs (Figure 2 a) show the terminal CO stretching vibration ν_(CO)_ at 1972 cm^−1^,^30^ which is different from those of Rh(CO)_2_(acac) (Figure S14) showing the symmetric and asymmetric *ν*_(CO)_ at 2062 cm^−1^ and 1993 cm^−1^, respectively.^31^ In addition, an absorption band at 442 cm^−1^ was observed for Rh‐P‐COFs (Figure 2 a), attributed to Rh‐P vibration, indicating a coordination bond between Rh and P.^32^ This was further confirmed by ^31^P static solid‐state NMR spectra for both Rh‐P‐COFs (Figure 2 c), which exhibited two new resonances at *δ*≈25 and 50 ppm, assigned to oxidized species P=O and P atoms coordinated with Rh, respectively.^33^ Furthermore, X‐ray photoelectron spectroscopy (XPS) spectra show that in the Rh 3d_5/2_ band (Figure 7 a) there are two peak at binding energy (BE) of 308.3 and 308.5 eV for Rh‐P‐COFs, which is slightly lower than for the parent Rh(CO)_2_(acac) (309.3 eV). The binding energies of the P 2p_3/2_ band in Rh‐P‐COFs (131.8 eV) are higher than that in P‐COFs (130.6 eV; Figure 7 b), attributed to electron transfer from P to Rh,18a, 33a resulting in more electron‐rich Rh atoms in the Rh‐P‐COFs. Meanwhile, the electron may also transfer from N to Rh, indicated by the binding energies of the N 1s band (Figure S15) for Rh‐P‐COFs (400.0 eV) and P‐COFs (398.9 eV). The peak at 132.5 eV (Figure 7 b) might be attributed to oxidized phosphorus atoms (P=O).^34^ Nevertheless, the current study could not illustrate the coordination mode of Rh with P‐COFs and the distribution of Rh (e.g., on crystal surface or in the matrix), which is very challenging and requires a further investigation with multiple techniques.

The Rh‐P‐COFs were employed as heterogeneous catalysts for hydroformylation of olefins^20^ under an optimized P/Rh ratio (Figure S16), reaction temperature (Figure S17), and reaction time (Figure S18). Initial experiments of hydroformylation of styrene were performed with two homogeneous catalysts, viz. Rh(CO)_2_(acac) and Rh(CO)_2_(acac) in combination with P(Ph)_3_. It was found (Table 1 ) that the latter indeed shows a higher turnover frequency (TOF, 3302 h^−1^) and aldehyde selectivity (96 %) than the only Rh(CO)_2_(acac) (1368 h^−1^ and 90 %, respectively). As expected, the heterogeneous Rh‐P‐COFs catalysts show higher TOFs (2557 h^−1^ for Rh‐P‐COF‐1 and 2074 h^−1^ for Rh‐P‐COF‐2, Table 1) and aldehyde selectivity (94 % for Rh‐P‐COF‐1 and 96 % for Rh‐P‐COF‐2, Table 1) than homogeneous Rh(CO)_2_(acac) catalyst. When prolonging the reaction time to 6 h, the olefin conversion over Rh‐P‐COFs reaches 95 % (Figure S18). Such promising catalytic conversion and product selectivity were also obtained for the hydroformylation of other olefins, for example, hex‐1‐ene, oct‐1‐ene, 4‐methoxystyrene, and 4‐chlorostyrene (Table S7). Furthermore, the reusability of the heterogeneous Rh‐P‐COFs catalysts were investigated by filtrating the used Rh‐P‐COFs catalysts and recycling several times. Similar olefin conversions after 6 h reaction was achieved over five cycles (Figure S19), with a small drop in aldehyde selectivity (Figure S19 and Table 1). Rh contents in the liquid products, analyzed by the inductively coupled plasma optical emission spectroscopy (ICP‐OES), indicate that there is no leaching of Rh active metal species. The limited decrease of the TOF for the 6th recycled Rh‐P‐COF‐1 (ca. 6.2 %) and Rh‐P‐COF‐2 (ca. 1.2 %) compared to the corresponding fresh Rh‐P‐COFs (Table 1) indicates an excellent reusability of the Rh‐P‐COFs catalysts.

**Table 1:** Hydroformylation of styrene over homogeneous Rh(CO)_2_(acac) without/with P(Ph)_3_ ligands and over the heterogeneous Rh‐P‐COFs catalysts.^[a]^

Catalyst	Conversion [mol %]	Selectivity of aldehydes [mol %]	Regioselectivity^[b]^	TOF^[c]^ [h^−1^]
Rh(CO)_2_(acac)	19	90	0.9	1368
Rh(CO)_2_(acac)+P(Ph)_3_	43	96	0.6	3302
Rh‐P‐COF‐1 (fresh)	34	94	0.9	2557
Rh‐P‐COF‐2 (fresh)	27	96	1.1	2074
Rh‐P‐COF‐1 (6th recycled)	31	97	1.1	2398
Rh‐P‐COF‐2 (6th recycled)	27	94	1.0	2050

[a] Reaction conditions: Rh dose 0.0023 mmol, molar ratio of P/Rh ca. 4.0, molar ratio of S/C (substrate/catalyst) of ca. 2000, CO/H_2_=1:1, *t*=0.25 h, *P*=2.0 MPa, *T*=100 °C, and 4 mL toluene. [b] Regioselectivity: molar ratio of linear (*n*‐) and branched (*iso*‐) aldehydes. [c]  TOF (Turnover Frequency) = N(Aldehydes)/(N(Rh) t(h)).

## Conclusions

In summary, we have demonstrated the synthesis of two examples of high crystalline and porous triphenylphosphine‐based covalent organic frameworks (P‐COFs) through a Schiff base reaction. Both P‐COF‐1 and P‐COF‐2 adopt AA stacking to form accessible open channels of 37 and 46 Å, respectively. The P‐COF‐supported Rh(CO)_2_(acac) catalysts (Rh‐P‐COFs) formed through Rh‐P coordination bonds have high turnover frequencies (>2000 h^−1^), high aldehyde selectivity (ca. 99 %), and excellent catalyst reusability (>5 cycles) for the hydroformylation of olefins. We believe that the novel triphenyl phosphine‐based COFs are not only suitable for immobilizing homogeneous metal‐based catalysts, but also have important potential applications in other fields such as adsorption–separation, electrocatalysis and photocatalysis.

## Conflict of interest

The authors declare no conflict of interest.

## Supporting information

As a service to our authors and readers, this journal provides supporting information supplied by the authors. Such materials are peer reviewed and may be re‐organized for online delivery, but are not copy‐edited or typeset. Technical support issues arising from supporting information (other than missing files) should be addressed to the authors.

Supplementary
