# Supplementary material for: Triphenylphosphine‐Based Covalent Organic Frameworks and Heterogeneous Rh‐P‐COFs Catalysts
Source: Chemistry. 2020 Aug 28;26(53):12134–9. doi: 10.1002/chem.202002150 (PMC7540510; doi:10.1002/chem.202002150)
Supplement: Supplementary file 1 — Supplementary [file CHEM-26-12134-s001.pdf]

Chemistry–A European Journal

Supporting Information

Triphenylphosphine-Based Covalent Organic Frameworks and Heterogeneous Rh-P-COFs Catalysts

Yubing Liu,[a] Alla Dikhtiarenko,[b] Naizhang Xu,[a] Jiawei Sun,[a] Jie Tang,[a] Kaiqiang Wang,[a] Bolian Xu,[a] Qing Tong,[a] Hero Jan Heeres,[c] Songbo He,*[c] Jorge Gascon,*[b] and Yining Fan*[a]

Table of contents

Section S1. General Information

Section S2. Synthetic Procedures

Section S3. Structural analysis and model refinement

Section S4. Hydroformylation reactions

Section S5. Supplementary Materials

Section S6. References

Section S1. General Information

1.1 Materials

Solvents were purified according to standard laboratory methods[1]. Other reagents were purchased from Sinopharm Chemical Reagent Co.Ltd, and used without further purification. Glass reaction tubes with volume of *ca*. 10 mL were dried before used. The internal pressure of the tubes was obtained using a Schlenk line.

1.2 Instrumentation

Liquid-state NMR spectra of ^{1}H, ^{13}C, and ^{31}P were recorded in $CDCl_3$ solution on a Bruker Avance III 400 MHz NMR spectrometer.

Elemental analysis was carried out on a Heraeus CHN-O-Rapid elemental analyzer.

The morphologies of samples were inspected using a field emission scanning electron microscopy (SEM, HITACHI S-4800) and transmission electron microscope (TEM, JEOL JEM-2100).

Powder X-ray diffraction (PXRD) data were collected on a Shimadzu Lab-X XRD-6000 using Cu Kα (λ=1.5406 Å) radiation operated at 40 kV and 30 mA with scanning speed of 1.0 degree min^{-1}.

The *in-situ* variable temperature PXRD (*In-situ* VT-PXRD) analyses were performed on D8 ADVANCE using a Cu Kα (λ= 1.5406 Å) radiation operated at 40 kV and 40 mA with scanning speed of 0.2 step s^{-1} at a temperature range of 30 °C to 300 °C under vacuum.

FT-IR spectra were taken on a Thermo Scientific Nicolet iS10 spectrometer.

Ultraviolet-visible (UV -vis) spectra were recorded with Shimadzu UV -3600 spectrophotometer.

The ^{13}C CP-MAS solid-state NMR spectra were recorded on a Bruker Avance III spectrometer with a 4-mm double-resonance MAS probe and with a sample spinning rate of 10.0 kHz. A contact time of 2 ms (ramp 100) and a pulse delay of 3 s were applied. The ^{31}P static solid-state NMR experiments were recorded on a Bruker Avance 500 spectrometer. $(NH_4)_2HPO_4$ was chosen as reference with ^{31}P chemical shift at 1.0 ppm.

The nitrogen adsorption and desorption isotherms were measured at 77 K using a Micromeritics ASAP 2020M system. The samples were degassed at 120 °C for 10 h before the measurements. The same protocol was also applied for the physorption of Ar at 87 K. The surface areas were calculated from the adsorption data using Brunauer-Emmett-Teller (BET) methods. The pore-size-distributions for the meso-pores and micro-pores were calculated from the N_2 adsorption using non-local density

functional theory (NLDFT) method[2].

X-ray photoelectron spectroscopy (XPS) spectra were performed on Thermo Fisher Scientific ESCALAB250Xi with Al Kα irradiation at θ = 0° for X-ray sources. The binding energies were calibrated using the C1 s peak at 284.6 eV.

Inductively coupled plasma optical emission spectroscopy (ICP-OES) analysis was recorded with PE Optima 5300DV.

Thermogravimetric and differential thermal analysis (TG/DTA) was recorded with Netzsch STA 449C from room temperature to 800 °C at a rate of 10 °C min^{-1} with a flow of N_2.

GC and GC-MS analyses were carried out over a Kejie GC5890C (Nanjing Kejie Analytical Instrument, Co. Ltd.) equipped with a SE-30 capillary chromatographic column and a Micromass GC-TOF equipped with a HP-5 capillary chromatographic column.

Section S2. Synthetic Procedures

2.1 Synthesis of precursors [3]

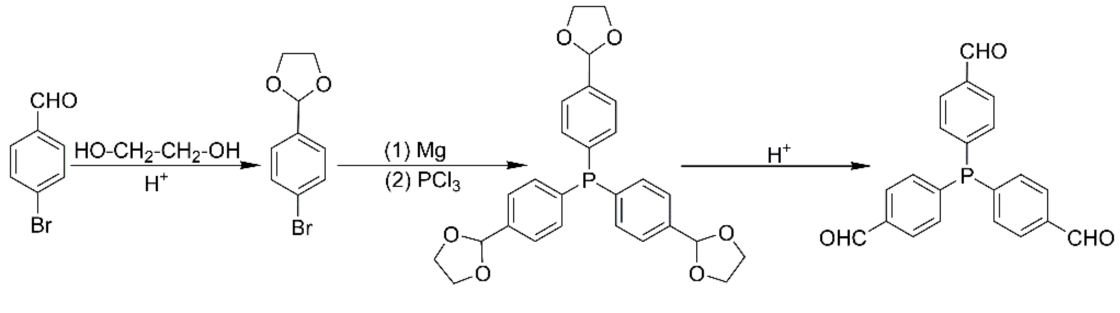

p-Bromobenzaldehyde (22 g, 0.12 mol), ethylene glycol (80 g), and *p*-toluene sulfonic acid monohydrate (2.0 g) were mixed with toluene (200 mL). The mixture was refluxed for 12 h. After reaction, the mixture was neutralized by adding $NaHCO_3$ aqueous solution, washed with NaCl aqueous solution, dried over $MgSO_4$, and evaporated under vacuum to give 2-(4-bromophenyl)-1,3-dioxolane (BD). Then, the Grignard reagent prepared from BD (23.6 g, 0.10 mol) and magnesium fine powders (2.76 g, 0.11 mol) in dry THF (150 mL) under sonication was cooled at 0 °C, and a solution of phosphorus trichloride (2.96 mL, 0.034 mol) in THF (10 mL) was then slowly added. The mixture was stirred for 12 h at room temperature and then was refluxed for 3 h. After cooling at 0 °C, a saturated $NaHCO_3$ aqueous solution (10 mL) was added. The mixture was extracted with CH_2Cl_2 (100 mL). The organic phase was washed with deionized water, dried on $MgSO_4$, and concentrated under reduced pressure. The white crystals *tris*[4-(1,3-dioxacyclopent-2-yl) phenyl] phosphane (TDCP) were crystallized from THF-ethanol (4:1) at 4 °C. A mixture of TDCP (10.6 g, 0.022 mol), 42 mL of 2 mol L^{-1} HCl aqueous solution and 150 mL THF were refluxed for 15 min and then cooled to room temperature and extracted with 100 mL CH_2Cl_2. The aqueous phase was neutralized with a saturated $NaHCO_3$ aqueous solution and extracted with CH_2Cl_2. The collected organic phases were washed with 5% $NaHCO_3$ aqueous solution and dried over $MgSO_4$. After distillation of the solvents, the crude semisolid residue was purified by chromatography (CH_2Cl_2, neutral alumina) to afford TFP as cream-colored crystals (5.1 g, 67% yield). ^1H NMR (400 MHz, $CDCl_3$) δ: 10.01 (s, 1H), 7.86 (dd, J = 8.1, 1.4 Hz, 2H), 7.45 (t, J = 7.7 Hz, 2H). ^{13}C NMR (101 MHz, $CDCl_3$) δ: 191.84 (s, 4H), 143.34 (d, J = 15.0 Hz, 1H), 137.06 (s, 1H), 134.43 (d, J = 19.9 Hz, 7H), 129.97 (d, J = 7.1 Hz, 7H). ^{31}P NMR (162 MHz, $CDCl_3$) δ: -3.9 ppm.

2.2 Synthesis of P-COF-1 and P-COF-2 materials

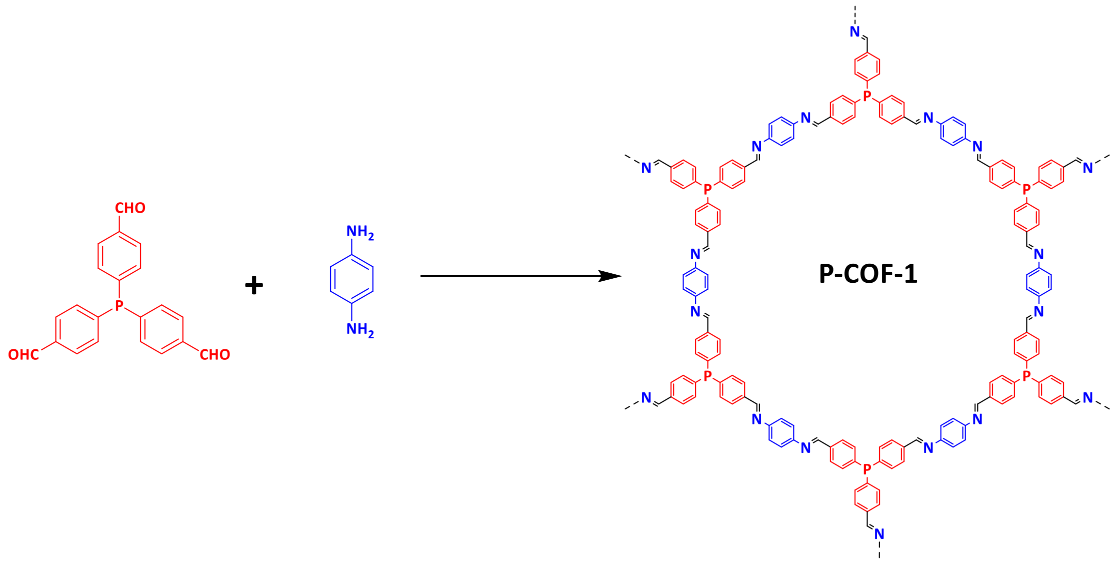

For the synthesis of P-COF-1, TFP (0.1039 g, 0.3 mmol) and *p*-phenylenediamine (0.0480 g, 0.45 mmol) were dissolved in 1,4-dioxane in a 10 mL glass tube. 0.3 mL of 6 mol L^{-1} aqueous acetic acid was added into the reaction mixture. Then the tube was flash frozen in a liquid nitrogen bath, evacuated to an internal pressure and then sealed. Upon warming to room temperature, the tube was placed in an oven at 130 °C and left undisturbed for 5 days. The yellow solid was isolated by centrifugation and washed with THF (3 × 10 mL). Further purification of the product was carried out by Soxhlet extraction in THF for 24 h, and then dried at 120 °C under vacuum for 12 h to yield P-COF-1 (0.0560 g, 40 % yield).

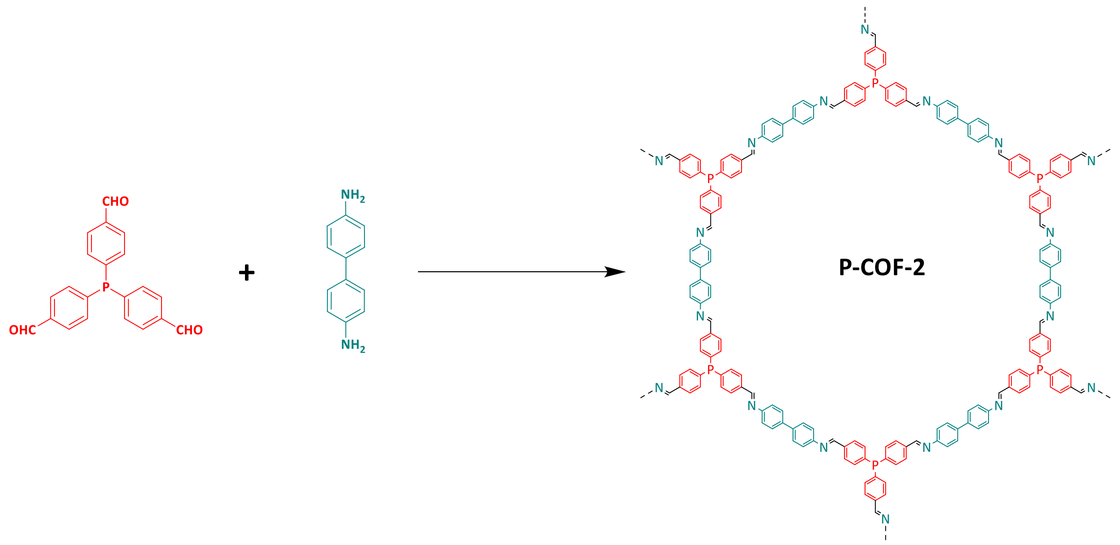

For the synthesis of P-COF-2, TFP (0.1039 g, 0.3 mmol) and benzidine (0.0818 g, 0.45 mmol) were dissolved in 1,4-dioxane and mesitylene in a 10 mL glass tube. The other procedures and conditions were the same as the synthesis of P-COF-1. Further purification of the product was carried out by Soxhlet extraction in THF for 36 h, and then dried at 120 °C under vacuum for 12 h to yield P-COF-2

(0.1320 g, 78 % yield).

The P-COF-1 and P-COF-2 were insoluble in water or common organic solvents such as acetone, ethanol, hexanes, N,N-dimethylformamide (DMF), and tetrahydrofuran (THF). Anal. Cald for ($C_{27}H_{19}N_2P$) n: C 87.3; H 5.2; N 7.5. Found: C 85.1; H 6.5; N 8.4. Anal. Cald for ($C_{33}H_{23}N_2P$) n: C 88.6; H 5.2; N 6.2. Found: C 87.6; H 5.3; N 7.1.

2.3 Synthesis of Rh-P-COFs catalysts

P-COFs (40 mg P-COF-1, 48 mg P-COF-2, *ca*. 0.1 mmol P) were pre-treated under vacuum at 120 °C for 2h. Dicarbonylacetylacetonato rhodium (I) (6.6 mg, 0.025 mmol) was dissolved in 1 mL of THF, and then the P-COF-1 or P-COF-2 was added at 50 °C under vacuum, respectively. The mixtures were kept at 50 °C for 2 h under stirring. The resulting solids were isolated by centrifugation and washed with THF, then dried at 50 °C under vacuum for 12 h to yield Rh-P-COFs. The Rh contents in Rh-P-COF-1 and Rh-P-COF-2 determined by ICP were 0.023 mmol (5.2 wt.%) and 0.025 mmol (4.7 wt.%), respectively.

2.4 Chemical Stability Test

P-COFs powder was suspended in different chemical environments for 24 h, including THF, DMF, ethanol, boiling water, HCl (aq) (pH=1), concentrated HCl (4 M), NaOH (aq) (pH=14), and concentrated NaOH (4M). The mixture was filtered and washed with excess water or methanol, and reactivated prior to characterization by PXRD.

Section S3. Structural analysis and model refinement

The structural models for P-COF-1 and P-COF-2 were built based on the structural units used in the synthesis procedure respecting their geometry and connectivity. The TFP was considered as tripodal fragment which could be connected by linear *p*-phenylenediamine or benzidine linkages to form extended 2D frameworks of P-COF-1 and P-COF-2. Corresponding structural unit for several proposed P-COF models were constructed based on these individual fragments and optimized applying geometrical energy minimization (GEM) procedure using Forcite molecular dynamics implemented in Accelrys Material Studio software package. The structural building unit adopted tetrahedral sp^3-hybridization configuration was introduced in the several unit cells: hexagonal $P6$ (AA-stacking) and $P6_3$ (AB-stacking), trigonal R-3 (ABC-stacking) and monoclinic Cc (slipped AB-stacking). These unit cells were calculated using Accelrys software previously optimized by Reflex module and used for Le Bail extraction and refinement. The refinement procedure included: background fit with six terms polynomial, peak shape modeled using Pearson VII function, unit cell parameters and zero-shifts. The profiles for all screened unit cells of corresponding structural models were calculated starting with the optimized unit cell parameters and the atomic positions.

Section S4. Hydroformylation reactions

The hydroformylation reactions were carried out in a 100 mL teflon-lined stainless-steel autoclave. The as-prepared Rh-P-COFs catalysts (4.6 mg Rh-P-COF-1, 5.1 mg Rh-P-COF-2, *ca*. 0.0023 mmol Rh), styrene (0.52 g, 5 mmol) and toluene (4 mL) were placed in the autoclave. After sealing and purging with N_2 for 3 times, the pressure of syngas (CO/H_2=1:1) was adjusted to the desired value. The reaction mixtures were stirred at 100 °C for 6 h. After reaction the catalyst was separated from the liquid phase of reaction mixture by centrifugation. The reaction products were analyzed by GC-FID and GC-MS equipped with SE-30 capillary column.

For catalyst recycling, the Rh-COFs catalysts were separated by centrifugation and washed with toluene in air. The catalyst was reused and the Rh content was analyzed by an inductively coupled plasma optical emission spectroscopy (ICP-OES) method.

Section S5. Supplementary Materials

Table S1. Crystallographic parameters and fractional atomic coordinates for P-COF-1 model arranged in A-A stacking mode.

Staking arrangement	**A-A** (eclipsed)	
Space group	hexagonal	
Crystal system	*P*6	
Unit cell parameters:	a = 32.549 Å	α = 90°
	b = 32.549 Å	β = 90°
	c = 7.9215 Å	γ = 120°

Fractional atomic coordinates

Atom labels	*x/a*	*y/b*	*z/c*
P1	−0.33344	0.33399	0.67897
C1	−0.36977	0.27044	0.65384
C2	−0.35829	0.24877	0.52437
H2	−0.33066	0.26699	0.45807
C3	−0.38724	0.20059	0.49239
H3	−0.37956	0.18590	0.40374
C4	−0.42760	0.17393	0.59115
C5	−0.43837	0.19511	0.72349
H5	−0.46537	0.17657	0.79235
C6	−0.40969	0.24331	0.75499
H6	−0.41713	0.25783	0.84477
C7	−0.45997	0.12273	0.55674
H7	−0.48587	0.10558	0.63148
N1	−0.45428	0.09624	0.41050
C8	−0.51842	−0.03037	0.55003
H8	−0.52812	−0.05026	0.66695
C9	−0.53117	−0.05410	0.39451
C10	−0.51832	−0.02778	0.24431
H10	−0.48326	0.04170	0.13266

Table S2. Crystallographic parameters and fractional atomic coordinates for P-COF-1 model arranged in A-B stacking mode.

Staking arrangement	**A-B** (staggered)	
Space group	hexagonal	
Crystal system	*P*6$_3$	
Unit cell parameters:	a = 32.549 Å	α = 90°
	b = 32.549 Å	β = 90°
	c = 15.843 Å	γ = 120°

Fractional atomic coordinates

Atom labels	*x/a*	*y/b*	*z/c*
P1	0.00776	0.99169	0.00746
C2	0.03429	0.95903	0.05179
C3	0.07752	0.96426	0.02325
H3	0.09484	0.98645	−0.02003
C4	0.09482	0.93690	0.05869
H4	0.12354	0.93965	0.03844
C5	0.07001	0.90533	0.12421
C6	0.02720	0.89999	0.15227
H6	0.01032	0.87831	0.19648
C7	0.00898	0.92648	0.11558
H7	−0.02063	0.92237	0.13400
C8	0.08996	0.87846	0.16625
H8	0.07305	0.85908	0.21296
N1	0.13517	0.87962	0.14035
C9	0.15159	0.85238	0.18496
C10	0.12575	0.82410	0.25369
H10	0.09323	0.82270	0.27341
C11	0.14181	0.79746	0.29731
H11	0.12151	0.77577	0.35027
C12	0.18385	0.79884	0.27263
C13	0.20969	0.82713	0.20390
H13	0.24222	0.82852	0.18417
C14	0.19364	0.85377	0.16027
H14	0.21393	0.87546	0.10731
N2	0.20027	0.77160	0.31723
P2	0.33469	0.66989	0.46492
C15	0.30614	0.69956	0.41632
C16	0.26446	0.69661	0.44814
H16	0.25043	0.67930	0.49839
C17	0.24392	0.71919	0.40586
H17	0.21524	0.71651	0.42621
C18	0.26544	0.74589	0.33338

C19	0.30653	0.74869	0.30169
H19	0.32088	0.76666	0.25216
C20	0.32674	0.72516	0.34259
H20	0.35451	0.72654	0.32029
C21	0.24548	0.77277	0.29134
H21	0.26240	0.79216	0.24465

Table S3. Crystallographic parameters and fractional atomic coordinates for P-COF-1 model arranged in A-B-C stacking mode.

Staking arrangement	**A-B-C**	(staggered-interpenetrated)
Space group	trigonal	
Crystal system	*R*-3	
Unit cell parameters:	a = 34.200 Å	α = 90°
	b = 34.200 Å	β = 90°
	c = 7.660 Å	γ = 120°

Fractional atomic coordinates

Atom labels	*x/a*	*y/b*	*z/c*
P1	0.33358	1.66553	1.37108
P2	0.33371	1.66652	1.37425
P3	0.33298	1.66624	1.36981
C1	0.46975	1.52138	1.51586
C2	0.36014	1.63246	1.37042
C3	0.40683	1.65441	1.39584
H3	0.42304	1.68583	1.42081
C4	0.42962	1.63035	1.38456
H4	0.46142	1.64532	1.40093
C5	0.40557	1.58410	1.34914
C6	0.35886	1.56186	1.32895
H6	0.34257	1.53021	1.30827
C7	0.33604	1.58588	1.33894
H7	0.30421	1.57077	1.32440
C8	0.42914	1.55778	1.33104
H8	0.43969	1.55580	1.21852
N1	0.44098	1.53607	1.48406
C9	0.47338	0.50043	0.34297
C10	0.50421	0.47969	0.33066
C11	0.53141	0.47990	0.49124
C12	0.52778	0.50085	0.66413
C13	0.49695	0.52159	0.67644

Table S4. Crystallographic parameters and fractional atomic coordinates for P-COF-2 model arranged in A-A stacking mode.

Staking arrangement	**A-A** (eclipsed)	
Space group	hexagonal	
Crystal system	*P*6	
Unit cell parameters:	a = 40.349 Å	α = 90°
	b = 40.349 Å	β = 90°
	c = 7.9215 Å	γ = 120°

Fractional atomic coordinates

Atom labels	*x/a*	*y/b*	*z/c*
P1	0.66731	0.33381	0.72843
C1	0.65247	0.36048	0.70281
C2	0.62574	0.35060	0.57282
H2	0.61735	0.32402	0.49321
C3	0.60935	0.37315	0.54035
H3	0.58773	0.36519	0.43354
C4	0.61950	0.40552	0.63913
C5	0.64527	0.41481	0.77197
H5	0.65273	0.44075	0.85460
C6	0.66189	0.39248	0.80397
H6	0.68314	0.40012	0.91213
C7	0.60336	0.43080	0.60422
H7	0.61047	0.45603	0.69102
N1	0.57730	0.42555	0.45745
C8	0.55486	0.44630	0.45222
C9	0.57353	0.48817	0.51174
C10	0.55065	0.50934	0.50641
C11	0.50911	0.48863	0.44156
C12	0.49044	0.44676	0.38203
C13	0.51332	0.42559	0.38736
H10	0.60428	0.50350	0.55975
H11	0.56447	0.54033	0.55048
H12	0.45968	0.43143	0.33402
H13	0.49950	0.39460	0.34330

Table S5. Peak assignment for the FT-IR spectrum of P-COF-1. [2, 5, 6]

Peak (cm^{-1})	Assignment and notes for P-COF-1
3026 (w)	Aromatic C-H stretch from phenyl rings
1694 (s)	Aldehyde C=O stretching
1614 (s)	Imine C=N stretching
1590 (s)	Aromatic C=C ring stretching from phenyl rings
1550 (m)	Aromatic C=C ring stretching from phenyl rings
1492 (s)	Aromatic C-C ring stretching vibration
1395 (m)	Aromatic ring stretching vibration aromatic C-C ring stretching vibration
1356 (m)	Aromatic ring bending vibration
1304 (m)	Aromatic ring stretching vibration
1271 (m)	Aromatic ring stretching vibration
1193 (s)	Imine C-C=N-C stretching. This mode is the stretching of the C-C and C-N single bonds
1174 (s)	C-Ph breathing
1102 (m)	C-Ph breathing
1087 (sh)<br>1014 (s)<br>969 (m)	Aromatic C-H in plane bending
876 (s)<br>833 (sh)	C-H out of plane bending vibration for *p*-substituted aromatic rings
815 (s)<br>719 (m)<br>670 (s)	Aromatic ring C-H out of plane bending vibration

w: weak; s: strong; sh: shoulder.

Table S6. Peak assignment for the FT-IR spectrum of P-COF-2. [2, 5, 6]

Peak (cm^{-1})	Assignment and notes for P-COF-1
3022 (w)	Aromatic C-H stretch from phenyl rings
1694 (s)	Aldehyde C=O stretching
1622 (s)	Imine C=N stretching
1589 (s)	Aromatic C=C ring stretching from phenyl rings
1550 (m)	Aromatic C=C ring stretching from phenyl rings
1484 (s)	Aromatic C-C ring stretching vibration
1399 (m)	Aromatic ring stretching vibration aromatic C-C ring stretching vibration
1359 (m)	Aromatic ring bending vibration
1306 (m)	Aromatic ring stretching vibration
1268 (m)	Aromatic ring stretching vibration
1197 (s)	Imine C-C=N-C stretching. This mode is the stretching of the C-C and C-N single bonds
1169 (s)	C-Ph breathing
1105 (m)	C-Ph breathing
1015 (s)<br>1002 (s)<br>969 (m)	Aromatic C-H in plane bending
883 (s)	C-H out of plane bending vibration for *p*-substituted aromatic rings
815 (s)<br>719 (m)<br>690 (s)	Aromatic ring C-H out of plane bending vibration

w: weak; s: strong.

Table S7. Hydroformylation of olefins over homogeneous $Rh(CO)_2(acac)$ without/with $P(Ph)_3$ ligands and heterogeneous Rh-P-COFs catalysts[a].

Entry	substrate	Catalyst	Conversion (mol%)	Aldehyde selectivity (mol%)	Regioselectivity[b]	Reaction time (h)
1	styrene	$Rh(CO)_2(acac)$	59	91	1.2	6
2		$Rh(CO)_2(acac)$ + $P(Ph)_3$	98	99	0.4	6
3		Rh-P-COF-1	95	99	1.3	6
4		Rh-P-COF-2	95	99	1.1	6
5	1-Hexene[c]	Rh-P-COF-1	94	87	1.1	6
6		Rh-P-COF-2	98	86	1.1	6
7	1-Octene[c]	Rh-P-COF-1	87	81	1.1	6
8		Rh-P-COF-2	92	85	1.1	6
9	4-Methoxystyrene	Rh-P-COF-1	94	92	0.7	6
10		Rh-P-COF-2	95	91	0.8	6
11	4-Chlorostyrene	Rh-P-COF-1	82	94	1.0	6
12		Rh-P-COF-2	94	98	1.0	6

[a]Reaction conditions: Rh dose 0.0023 mmol, molar ratio of P/Rh *ca.* 4.0, molar ratio of S/C (substrate/catalyst) of *ca.* 2000, $CO:H_2$ = 1:1, P = 2.0 MPa, T = 100 °C, 4 mL toluene and reaction time of 6 h. [b]Regioselectivity: molar ratio of linear (*n*-) aldehydes/branched (*iso*-) aldehydes. [c]P = 3.0 MPa.

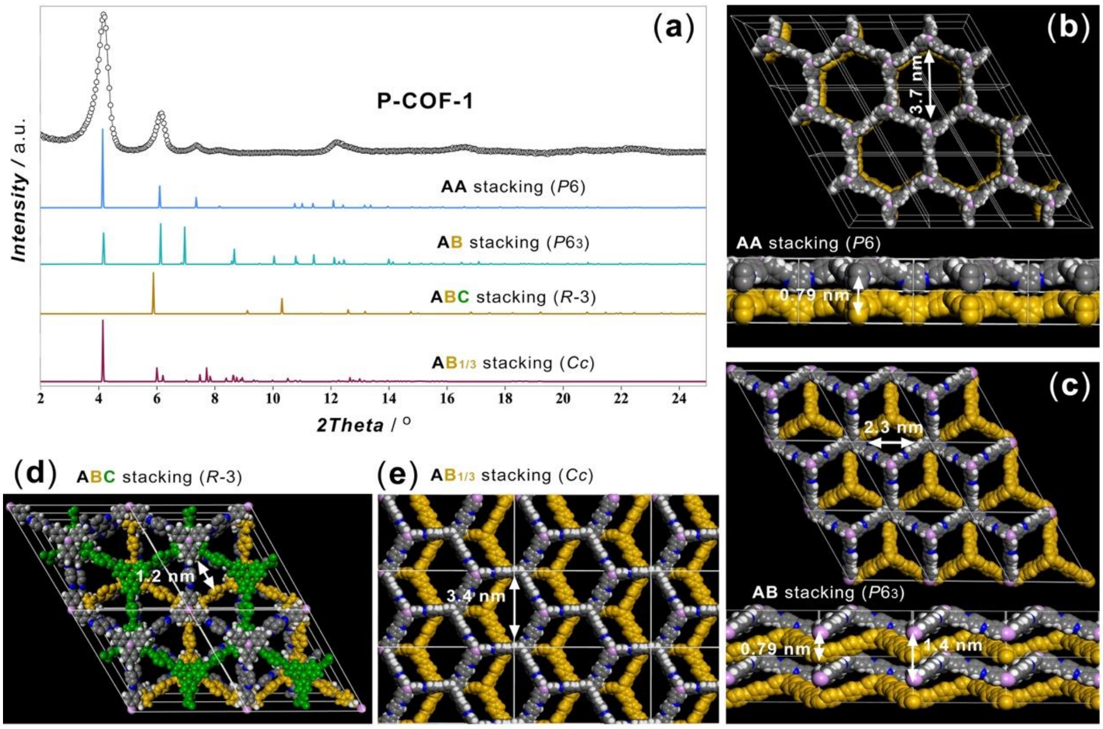

Figure S1. Powder X-ray diffraction pattern of P-COF-1 (black dots) compared to the simulated for AA eclipsed (blue curve, *P*6 space group), AB staggered (turquoise, $P6_3$), ABC staggered-interpenetrated (orange, *R*-3) and $AB_{1/3}$ partially staggered (purple, *Cc*) models. The structural arrangement of AA model (b) with open pore of 3.2 nm and interlayer distance of 0.79 nm, AB model (c) with window opening of 2.3 nm and interlayer distance of 0.79 nm (double layer of 1.4 nm), ABC model (d) with window opening of 1.2 nm and $AB_{1/3}$ partially staggered with uniform windows of 3.4 nm size in P-COF-1 structures viewed along *c*-axes.

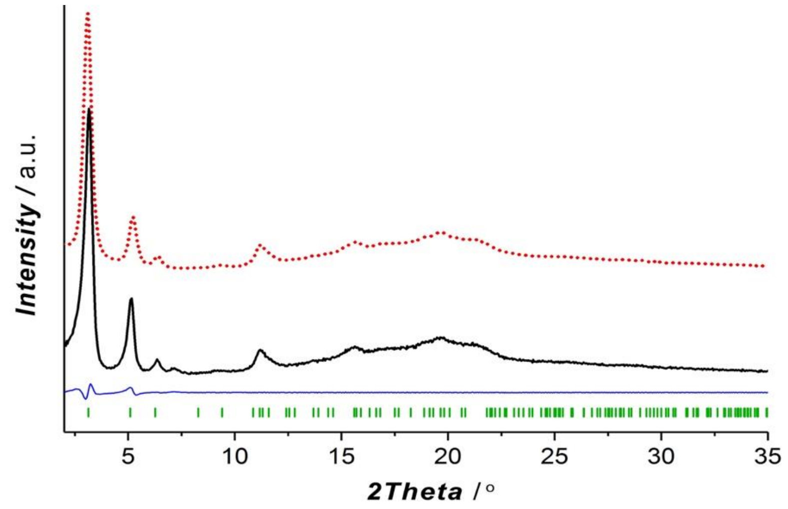

Figure S2. Le Bail fitting plot of P-COF-1 with respect to AA-stacked model of hexagonal *P*6 space group. Green sticks - Bragg positions, black solid line - experimental pattern, red dotted line - result of refinement and blue solid line is difference curve. Refinement statistics: R_p = 7.24%, R_{wp} = 12.86%.

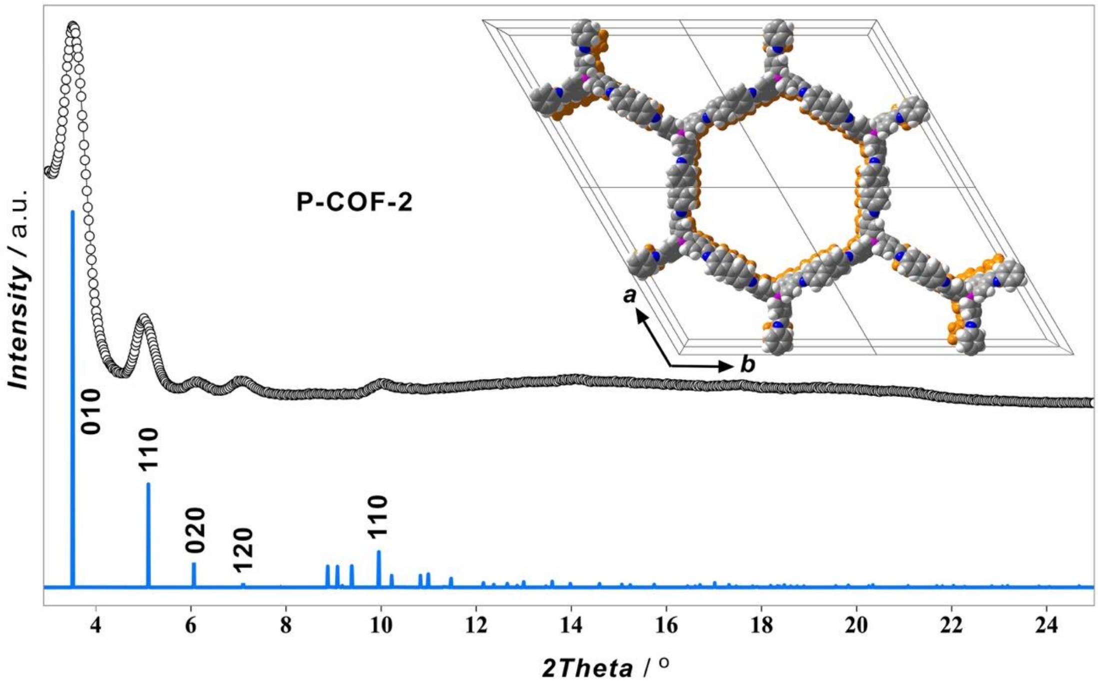

Figure S3. Powder X-ray diffraction pattern of P-COF-2 compared to the simulated from AA-stacked model constructed in hexagonal settings applying *P*6 space group.

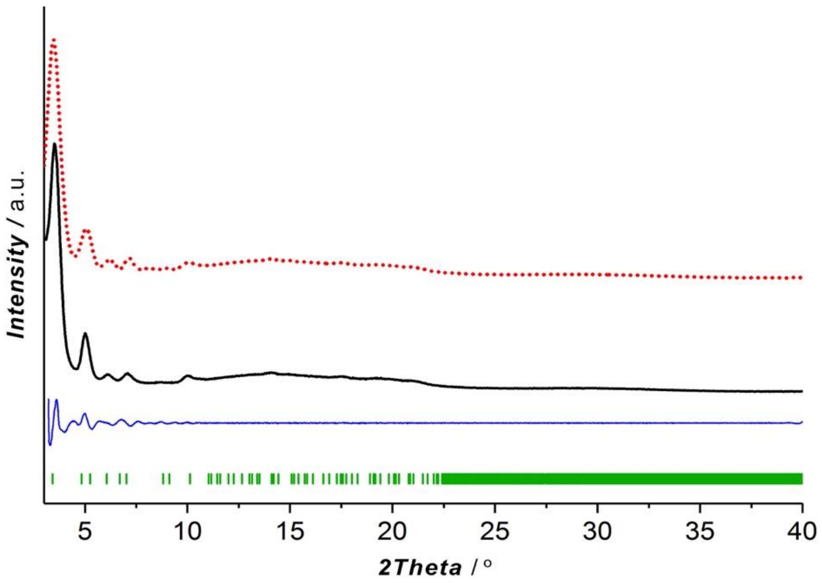

Figure S4. Le Bail fitting plot of P-COF-2 with respect to the AA-stacked model with hexagonal settings of *P*6 space group. Green sticks correspond to the Bragg positions, black solid line - experimental pattern, red dotted line - result of refinement and blue solid line is difference curve. Refinement statistics: R_p = 4.02%, R_{wp} = 6.20%.

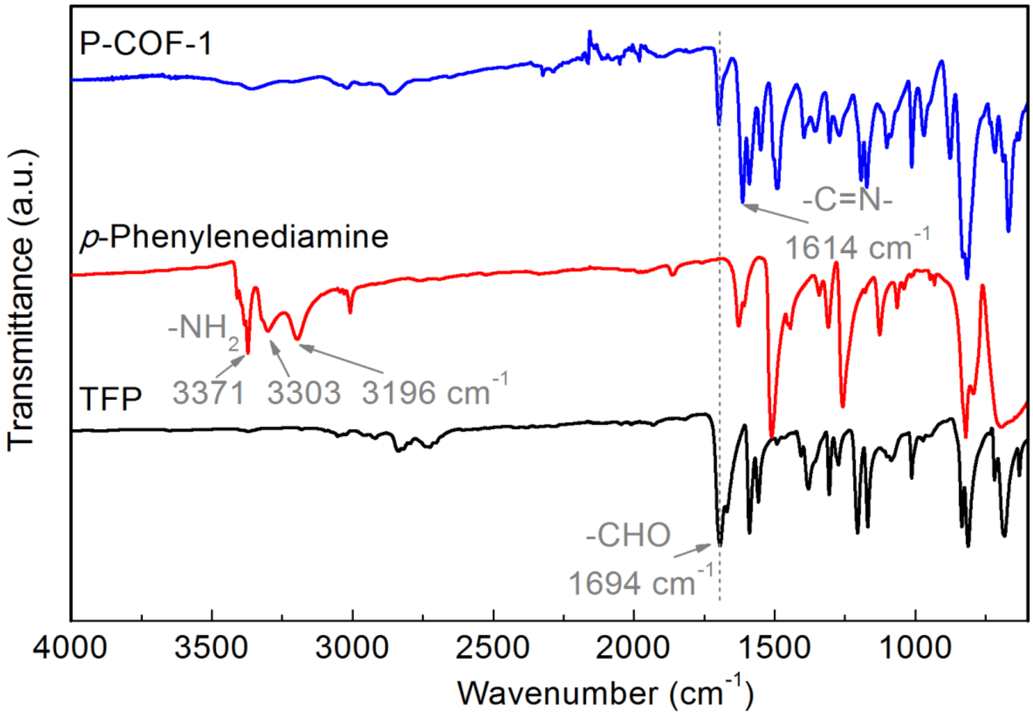

Figure S5. FT-IR spectra of TFP, *p*-phenylenediamine and P-COF-1.

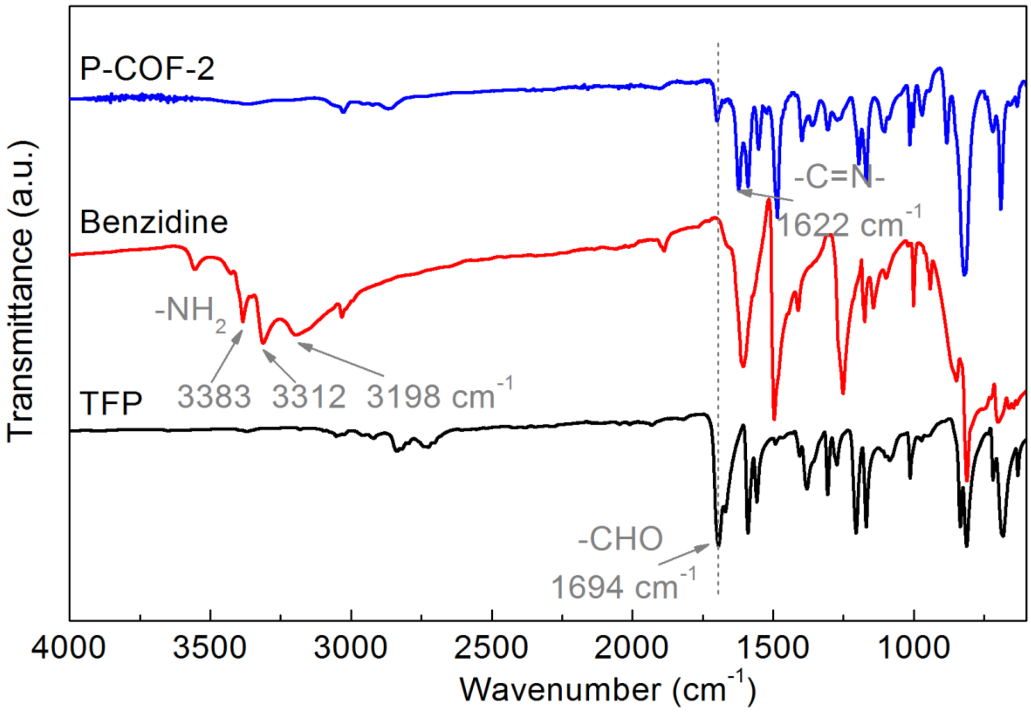

Figure S6. FT-IR spectra of TFP, benzidine and P-COF-2.

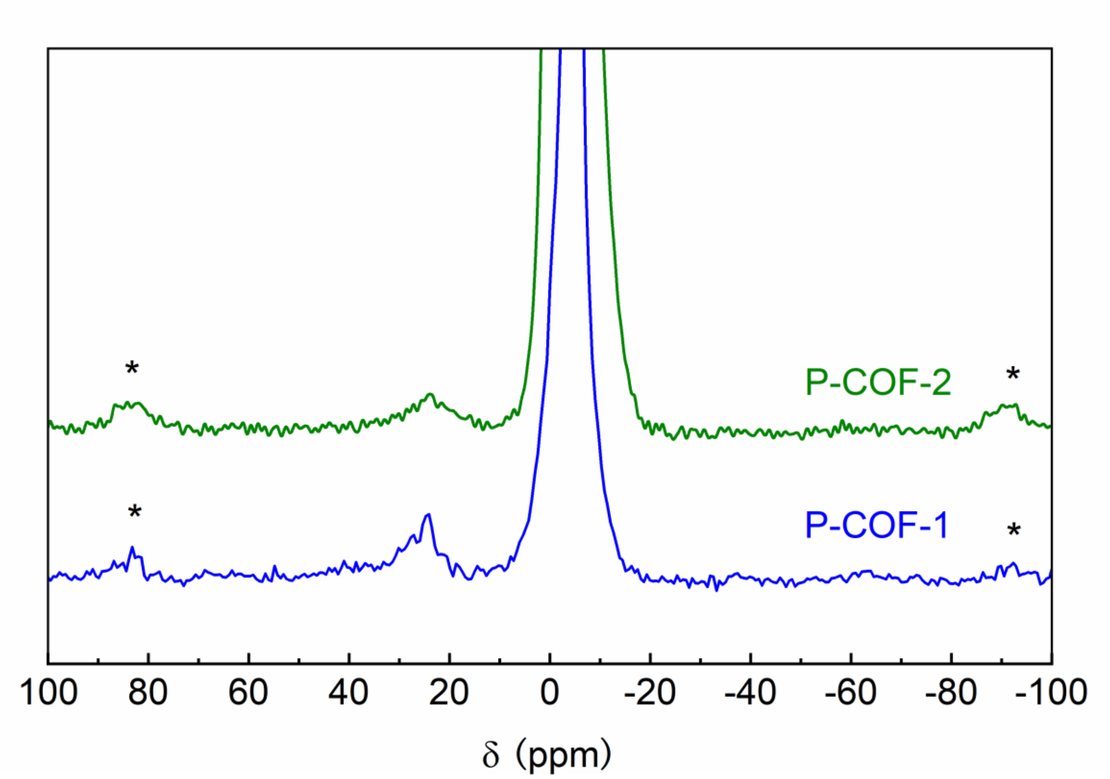

Figure S7. Enlarged NMR spectra of P-COF-1 and P-COF-2.

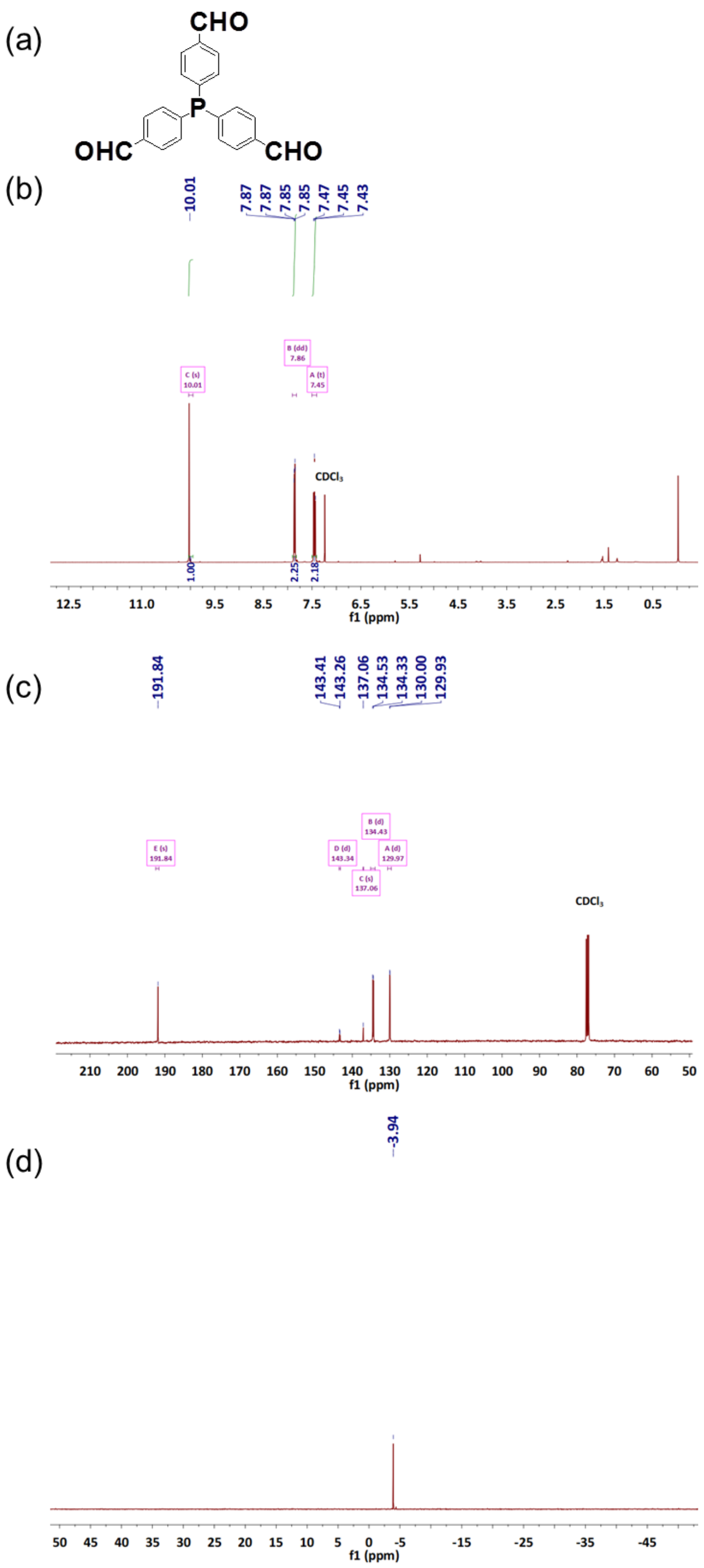

Figure S8. The TFP structural formula (a) and liquid-state NMR spectra of TFP (b) ^{1}H, (c) ^{13}C and (d) ^{31}P.

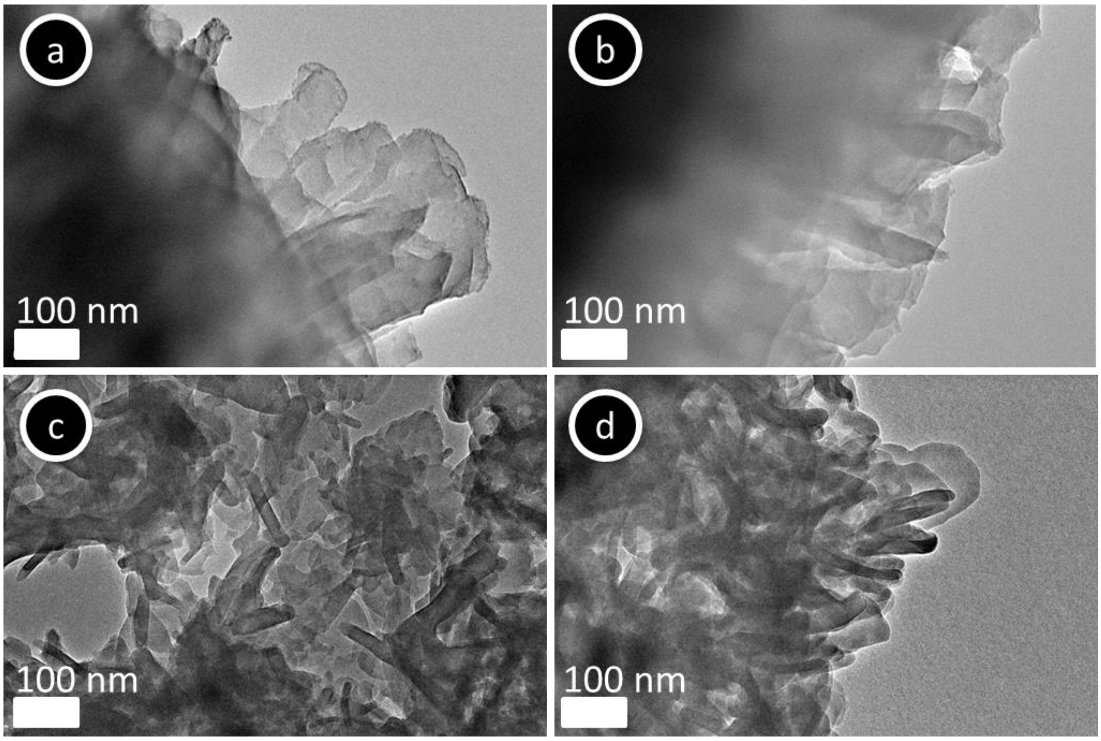

Figure S9. TEM images of (a) P-COF-1, (b) Rh-P-COF-1, (c) P-COF-2 and (d) Rh-P-COF-2.

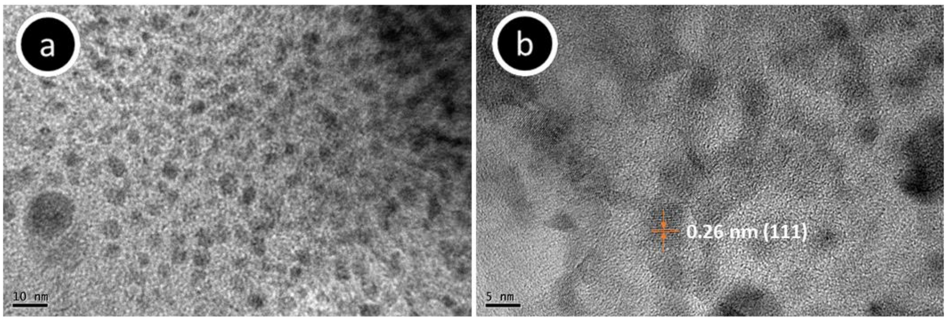

Figure S10. HR-TEM of Rh-P-COF-1 reduced by $NaBH_4$ under different magnifications.

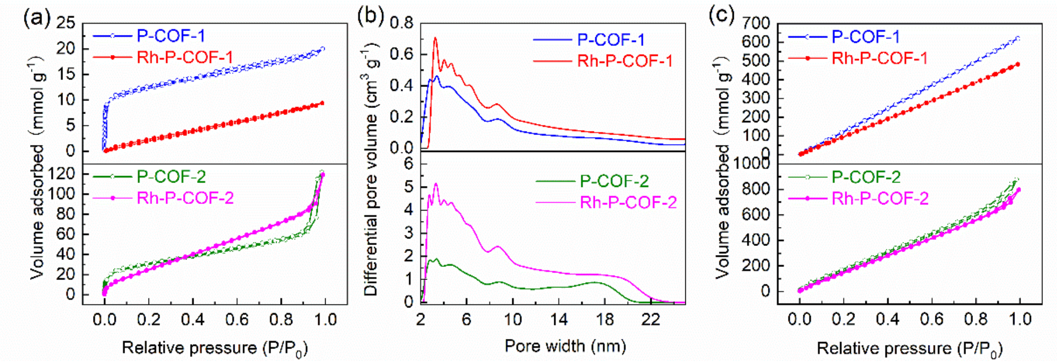

Figure S11. (a) Nitrogen adsorption/desorption isotherms, (b) DFT pore size distribution from N_2 adsorption of P-COFs and Rh-P-COFs, and (c) Ar adsorption/desorption isotherms.

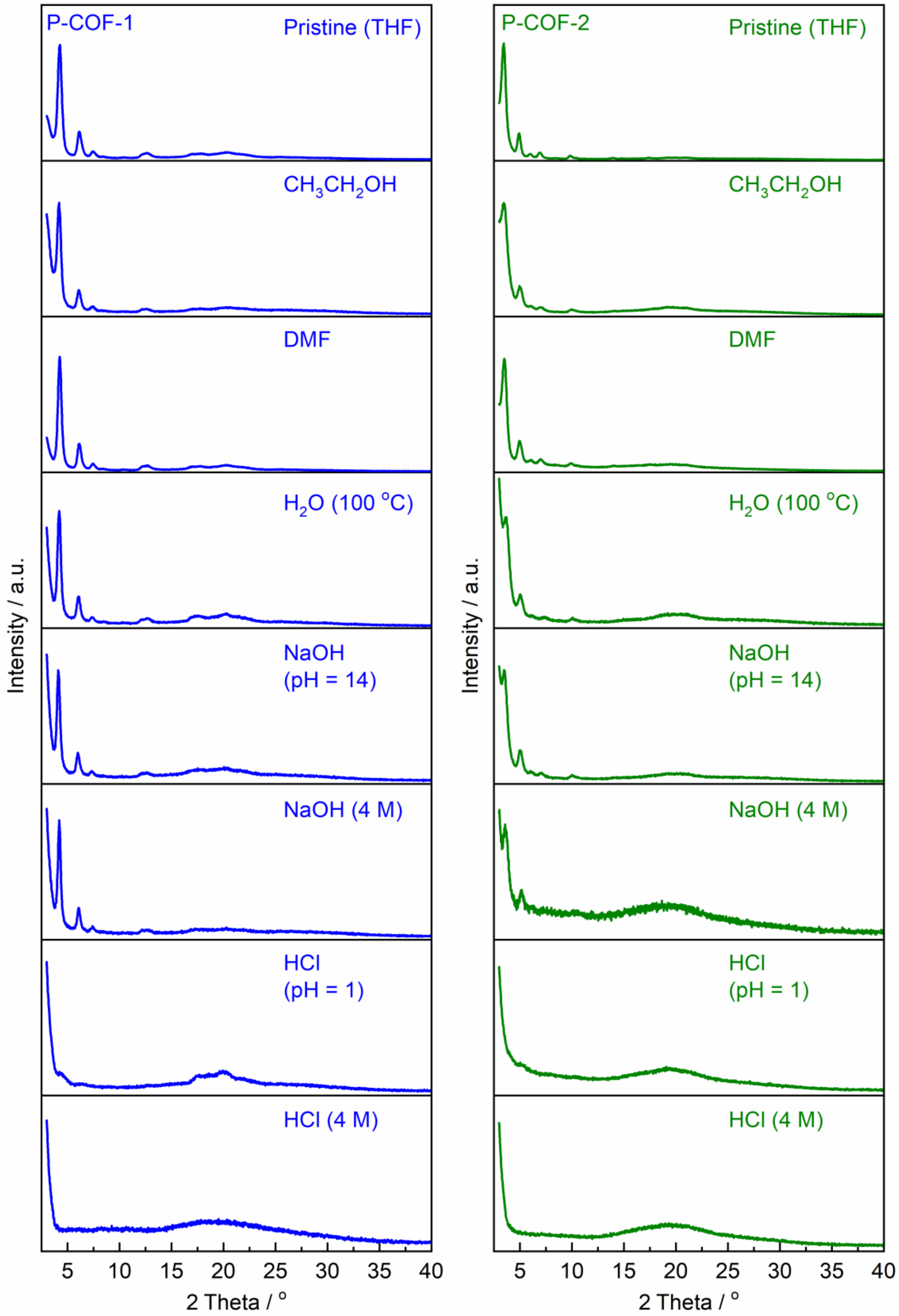

Figure S12. PXRD patterns of P-COFs after the treatment with different solvent for 24 h.

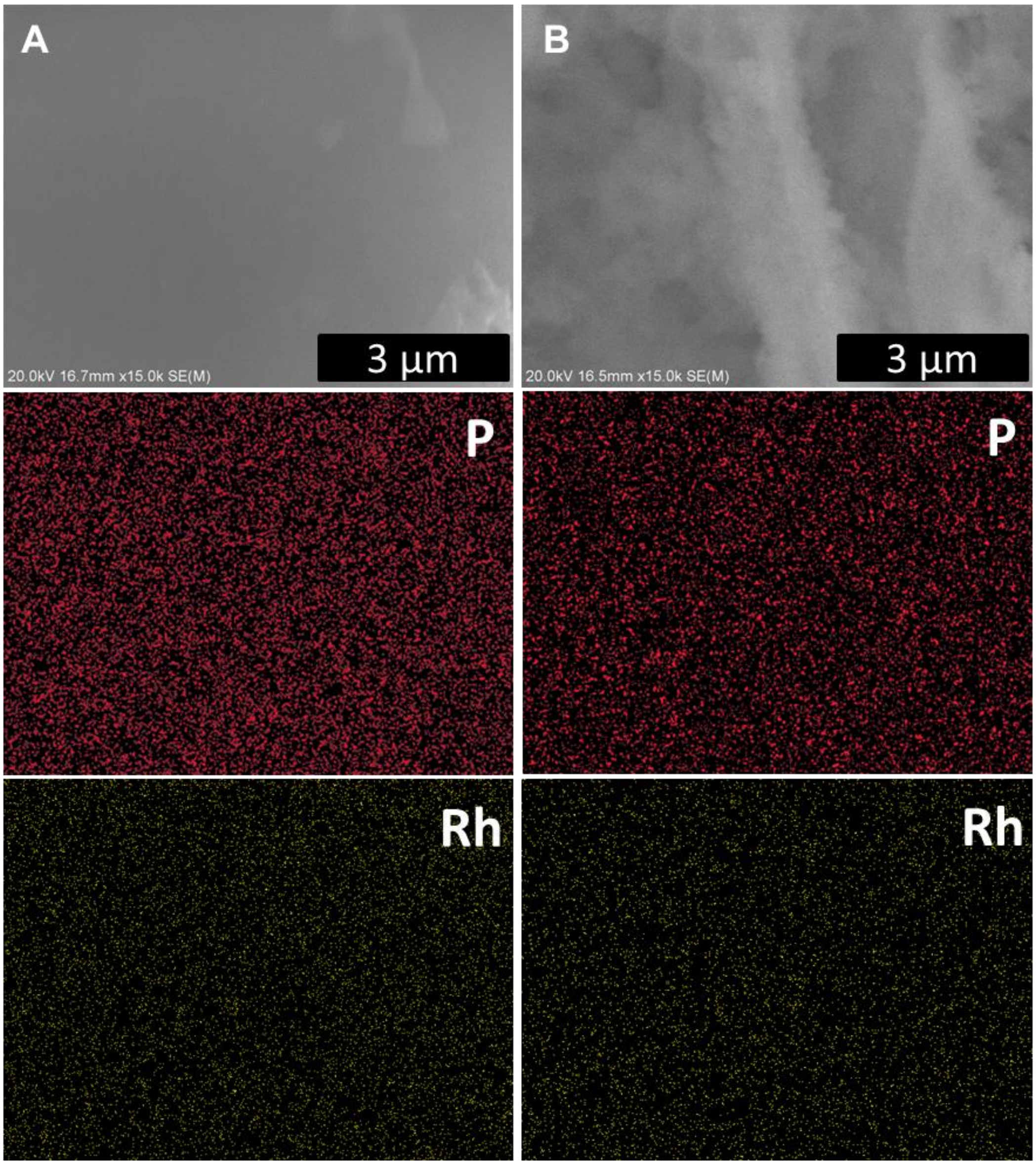

Figure S13. SEM-EDS mapping of (A) Rh- P-COF-1 and (B) Rh- P-COF-2.

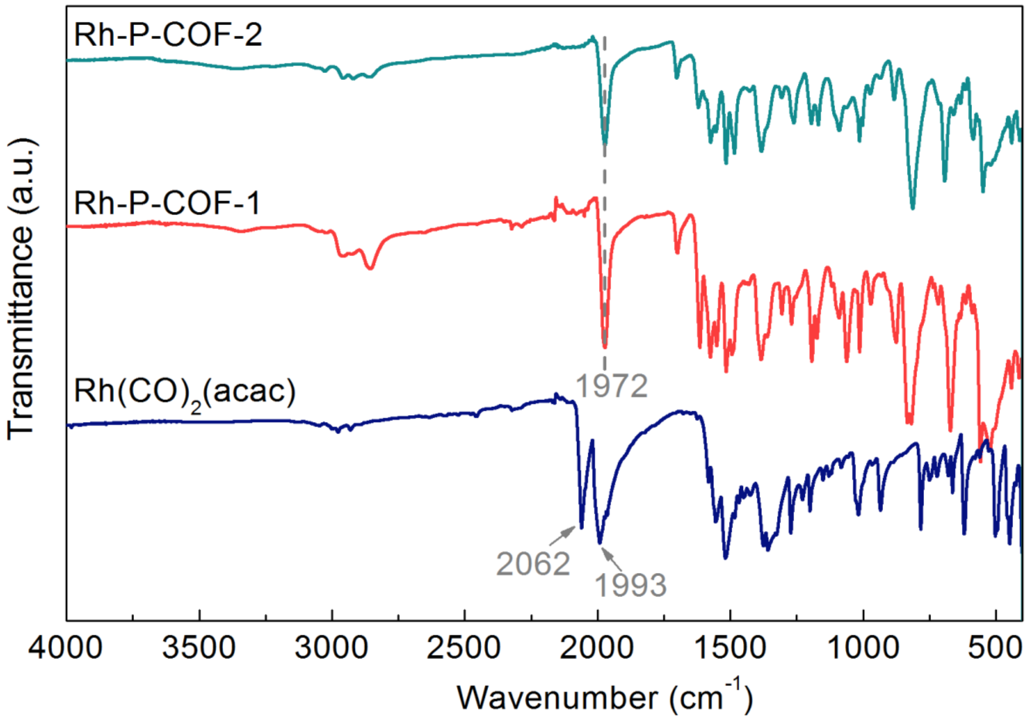

Figure S14. FT-IR spectra of $Rh(CO)_2(acac)$ and Rh-P-COFs.

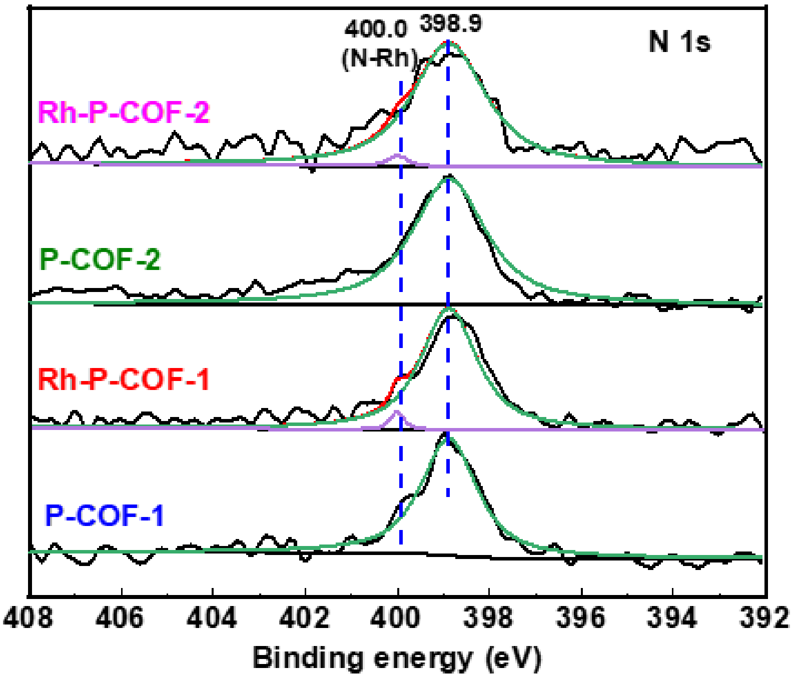

Figure S15. XPS of N 1s for P-COFs and Rh-P-COFs.

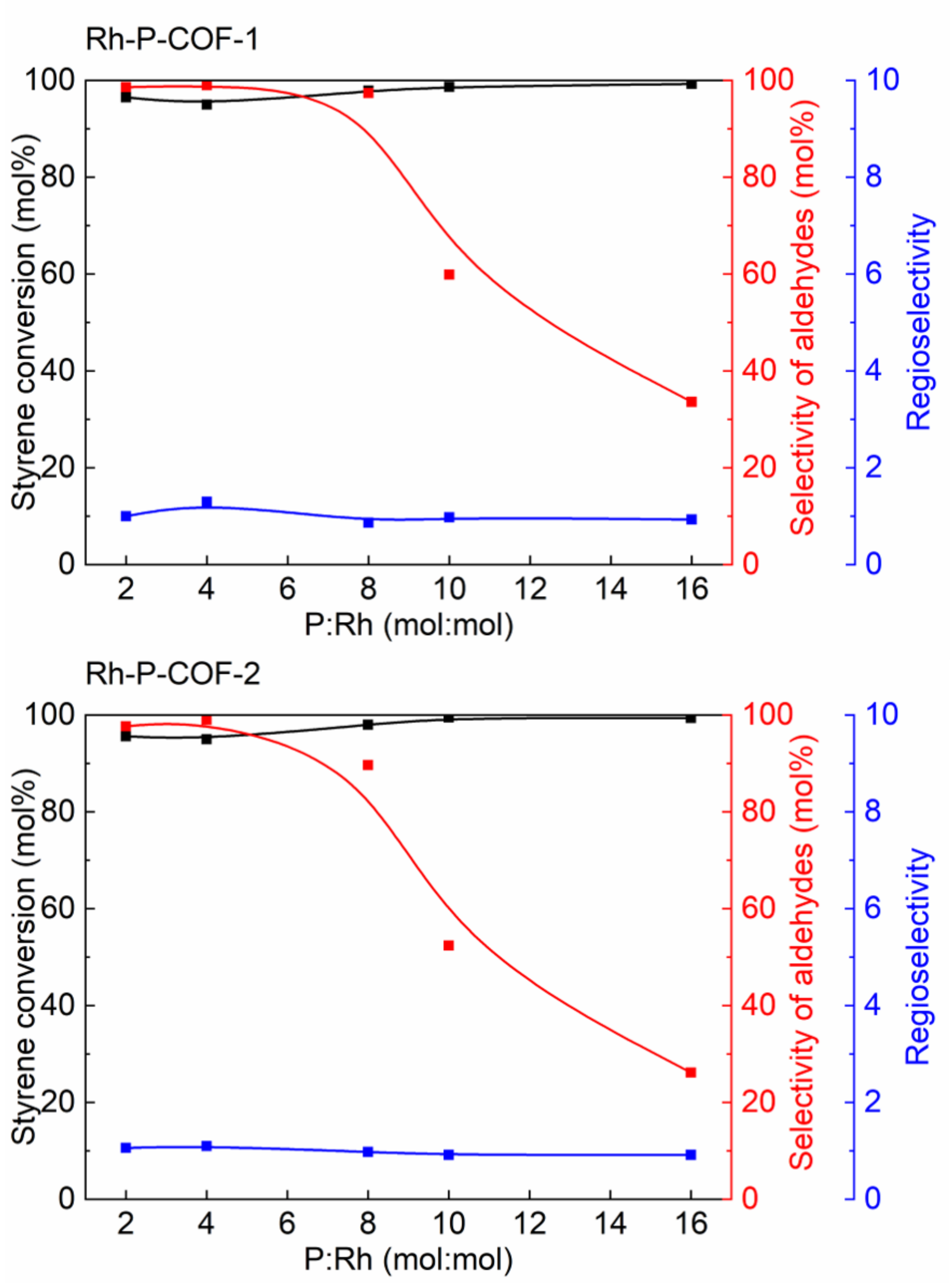

Figure S16. Hydroformylation of styrene over the Rh-P-COFs catalyst *versus* P/Rh ratio.

Reaction conditions: Rh dose 0.0023 mmol, molar ratio of S/C (substrate/catalyst) of ca. 2000, CO:H_2 = 1:1, P = 2.0 MPa, T = 100 °C, 4 mL toluene and reaction time of 6 h.

Regioselectivity: molar ratio of linear (*n*-) aldehydes/branched (*iso*-) aldehydes.

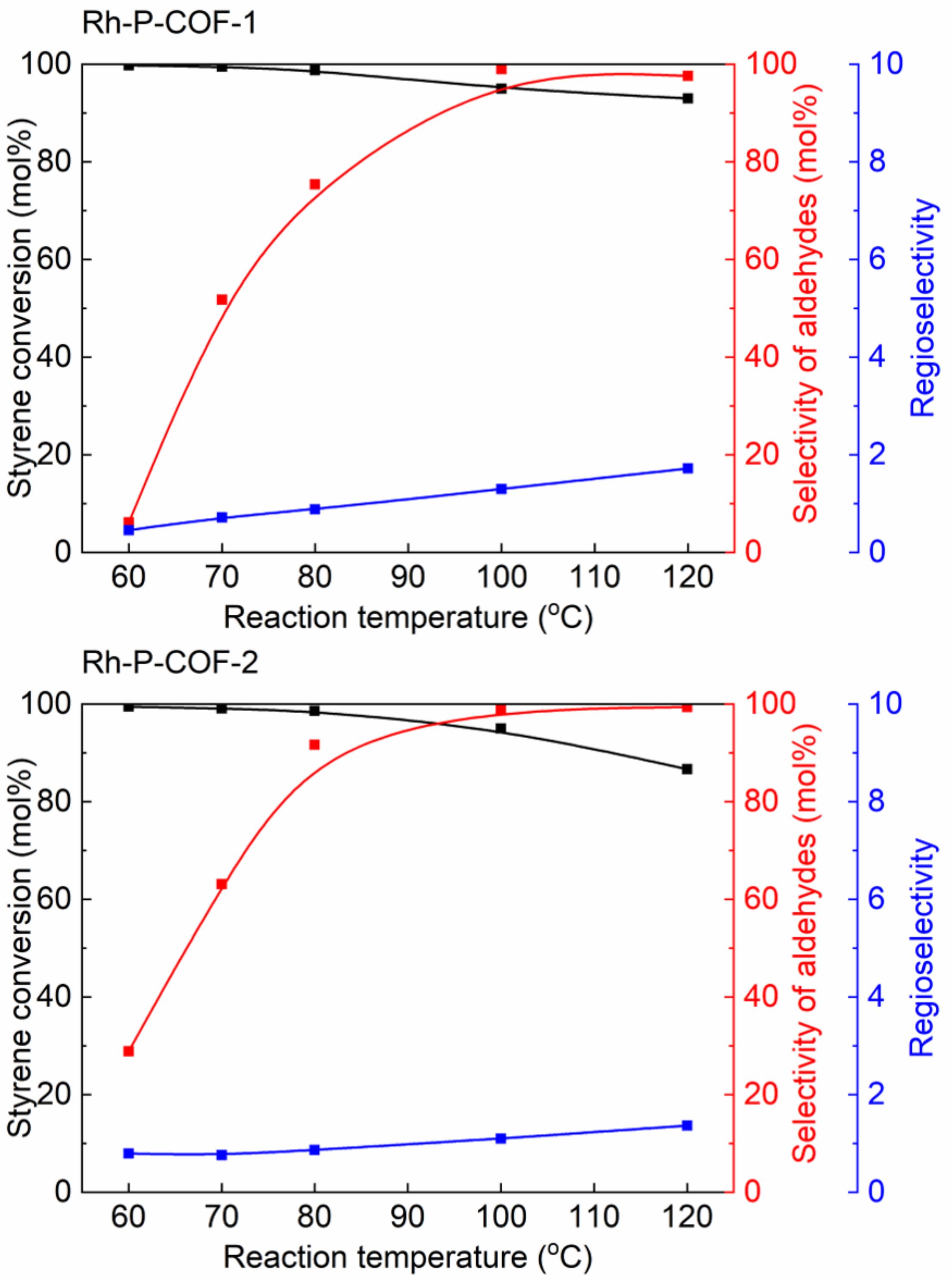

Figure S17. Hydroformylation of styrene over the Rh-P-COFs catalyst *versus* reaction temperature.

Reaction conditions: Rh dose 0.0023 mmol, molar ratio of P/Rh ca. 4.0, molar ratio of S/C (substrate/catalyst) of ca. 2000, $CO:H_2$ = 1:1, P = 2.0 MPa, 4 mL toluene and reaction time of 6 h.

Regioselectivity: molar ratio of linear (*n*-) aldehydes/branched (*iso*-) aldehydes.

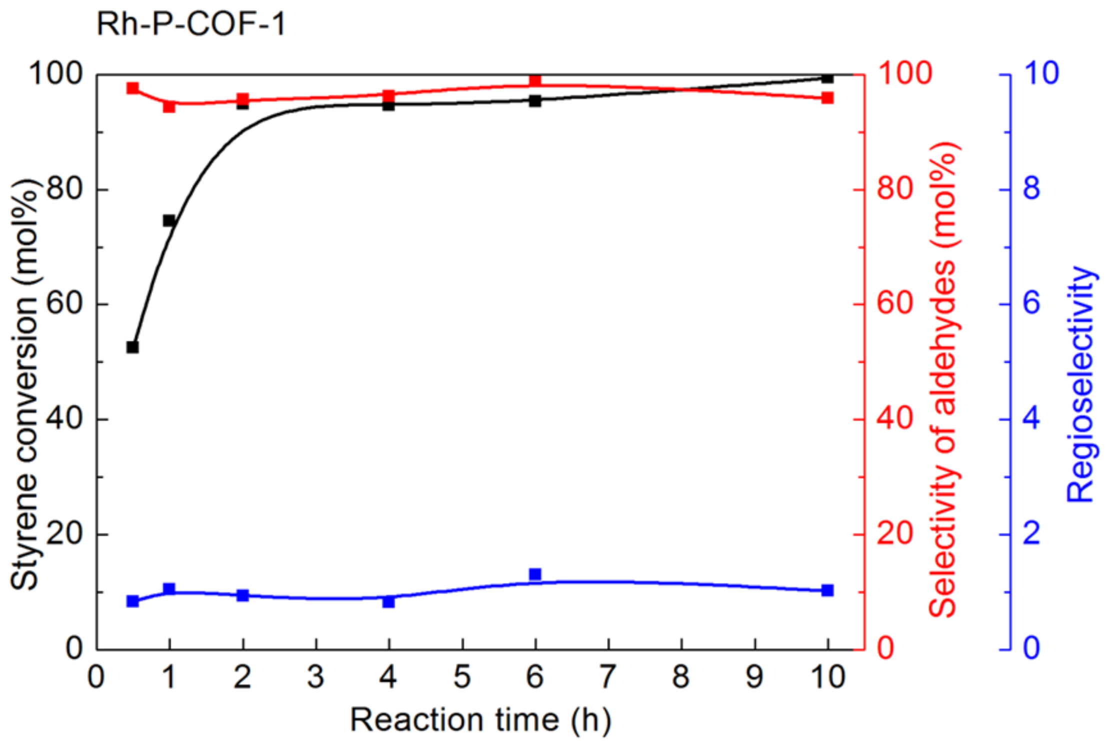

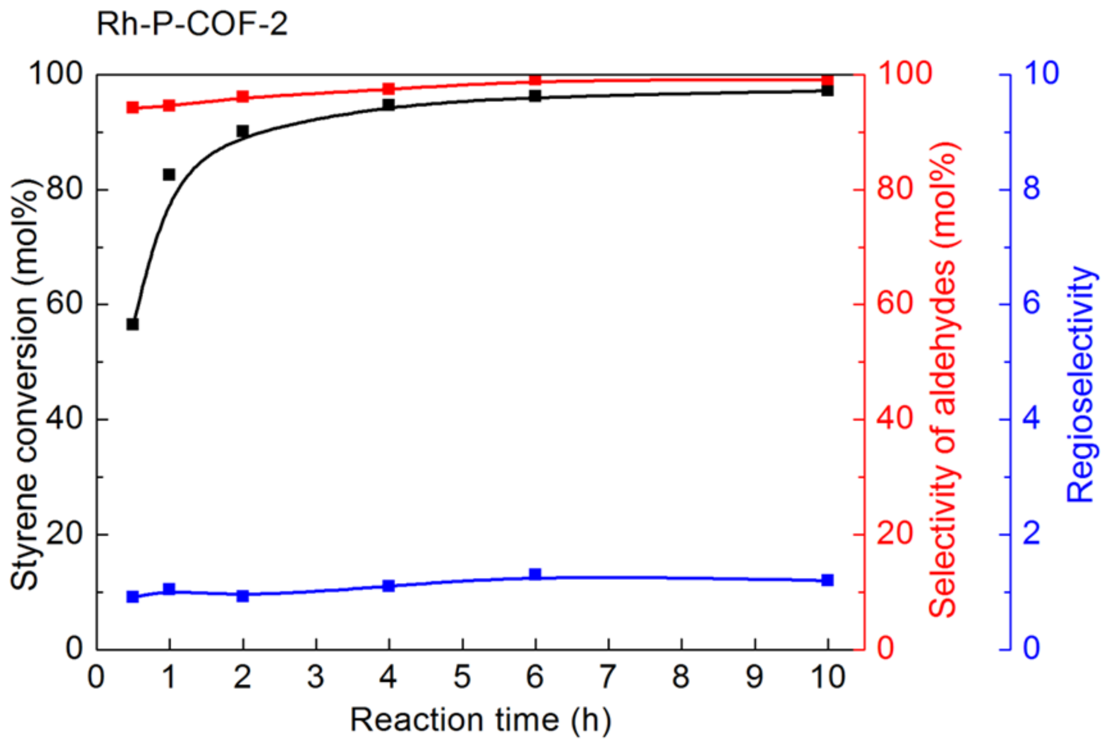

Figure S18. Hydroformylation of styrene over the Rh-P-COFs catalyst *versus* reaction time.

Reaction conditions: Rh dose 0.0023 mmol, molar ratio of P/Rh ca. 4.0, molar ratio of S/C (substrate/catalyst) of ca. 2000, CO:H_2 = 1:1, P = 2.0 MPa, T = 100 °C, 4 mL toluene.

Regioselectivity: molar ratio of linear (*n*-) aldehydes/branched (*iso*-) aldehydes.

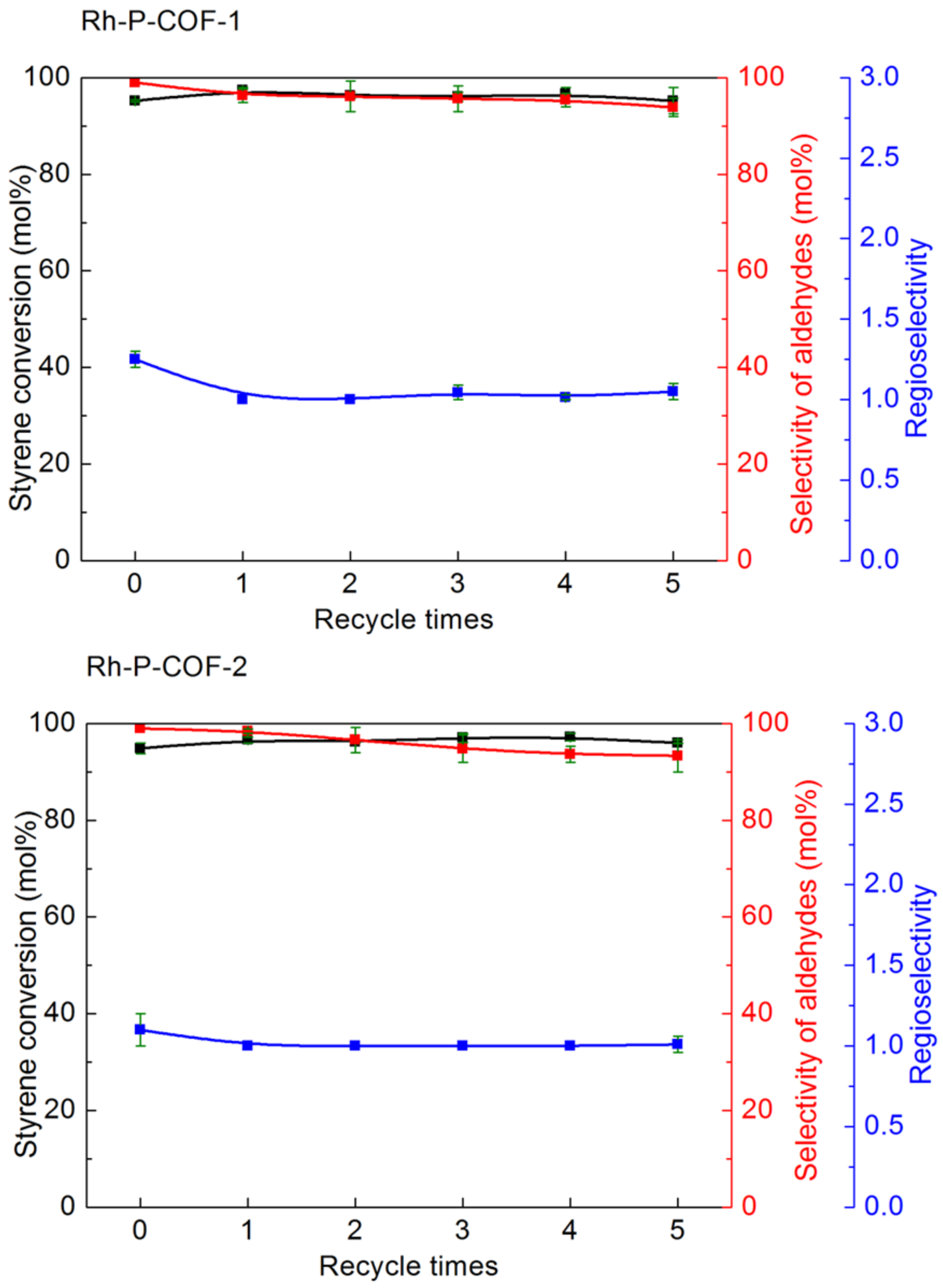

Figure S19. Reusability of Rh-P-COFs heterogenous catalysts for hydroformylation of styrene.

Reaction conditions: Rh dose 0.0023 mmol, molar ratio of P/Rh *ca*. 4.0, molar ratio of S/C (substrate/catalyst) of *ca*. 2000, CO:H_2 = 1:1, P = 2.0 MPa, T = 100 °C, 4 mL toluene and reaction time of 6 h for each cycle.

Regioselectivity: molar ratio of linear (*n*-) aldehydes/branched (*iso*-) aldehydes.

Section S6. References

[1] W. L. F. Armarego, C. Chai, W. L. F. Armarego, C. Chai, *Purification of Laboratory Chemicals (6th Edition)*, Butterworth-Heinemann, Linacre House, Jordan Hill, Oxford OX2 8DP, UK, **2009**.

[2] F. J. Uribe-Romo, J. R. Hunt, H. Furukawa, C. Klock, M. O'Keeffe, O. M. Yaghi, *J. Am. Chem. Soc.* **2009**, *131*, 4570-4571.

[3] a) H. Nishi, T. Namari, S. Kobatake, *J. Mater. Chem.* **2011**, *21*, 17249-17258; b) F. Chalier, Y. Berchadsky, J.-P. Finet, G. Gronchi, S. Marque, P. Tordo, *J. Phys. Chem.* **1996**, *100*, 4323-4330.

[5] a) P. Albacete, J. I. Martínez, X. Li, A. López-Moreno, S. a. Mena-Hernando, A. E. Platero-Prats, C. Montoro, K. P. Loh, E. M. Pérez, F. Zamora, *J. Am. Chem. Soc.* **2018**, *140*, 12922-12929; b) S. Wan, J. Guo, J. Kim, H. Ihee, D. Jiang, *Angew. Chem., Int. Ed.* **2008**, *47*, 8826-8830.

[6] H. G. M. Edwards, A. F. Johnson, I. R. Lewis, *Spectrochim. Acta, Part A.* **1993**, 49, 707-714.
